# METTL3 promotes neutrophil extracellular trap formation via SYK/ERK/MEK signaling during acute lung injury

**DOI:** 10.1016/j.jare.2026.01.044

**Published:** 2026-01-22

**Authors:** Shuhua Luo, Qianqian Zhang, Wending Zhou, Lina Zhang, Chaoxiong Liao, Dan Zeng, Liangqing Zhang, Fu-li Xiang, Jie Xu, Jing Tang

**Affiliations:** a Department of Anesthesiology, Affiliated Hospital of Guangdong Medical University, Zhanjiang, Guangdong, China; b Guang Dong Medical University, Zhanjiang, Guangdong, China; c Department of Anesthesiology, the Second Affiliated Hospital of Guangdong Medical University, Zhanjiang, Guangdong, China; d Institute of Precision Medicine, First Affiliated Hospital of Sun Yat-sen University, Guangzhou, China; e National-Guangdong Joint Engineering Laboratory for Diagnosis and Treatment of Vascular Diseases, Sun Yat-sen University, Guangzhou, China; f NHC Key Laboratory of Assisted Circulation and Vascular Diseases, Sun Yat-sen University, Guangzhou, China

**Keywords:** METTL3, m6A, Neutrophil extracellular trap, SYK, ALI

## Abstract

**Introduction:**

Acute lung injury (ALI) is a life-threatening condition characterized by overwhelming pulmonary inflammation and neutrophil infiltration. Neutrophils play a central role in ALI pathogenesis by releasing proteolytic enzymes, reactive oxygen species, and neutrophil extracellular traps (NETs). Although NETs contribute to pathogen clearance, excessive NET formation exacerbates tissue injury and amplifies inflammation. N6-methyladenosine (m6A) is the most abundant internal modification of eukaryotic mRNA and plays a pivotal role in immune cell regulation by modulating RNA stability and translation. However, how m6A modification governs NETosis during ALI remains incompletely understood.

**Objectives:**

This study aimed to investigate the role of METTL3 mediated m6A modification on NET formation and the underlying mechanisms in lung injury in ALI.

**Methods:**

The role of METTL3 mediated m6A in ALI was examined in mouse with conditional deletion of METTL3 in myeloid or neutrophil lineages. Histological and flow cytometric analysis, gene interference, overexpression, transcriptomic profiling and m6A-RIP-qPCR were performed to elucidate the underlying mechanisms of METTL3 promoting NETosis via SYK/ERK/MEK signaling axis in vitro and in vivo.

**Results:**

Conditional deletion of METTL3 in myeloid or neutrophil lineages significantly reduced lung MPO activity, citrullinated histone H3 levels, and extracellular DNA deposition following lipopolysaccharide (LPS)-induced ALI. METTL3 deficiency attenuated pulmonary inflammation and reduced neutrophil infiltration. In vitro, METTL3-deficient neutrophils exhibited decreased NET formation upon phorbol myristate acetate (PMA) stimulation, along with diminished SYK phosphorylation and downstream ERK/MEK activation. Mechanistically, METTL3 promotes m6A methylation of SYK mRNA, enhancing its stability and transcription. Pharmacological inhibition of either METTL3 or SYK recapitulated the protective phenotype, suppressing NETosis and lung damage in vivo.

**Conclusion:**

Collectively, our findings uncover a novel METTL3–SYK epitranscriptomic axis that governs neutrophil effector responses during endotoxin-induced lung injury. These results highlight m6A-dependent post-transcriptional regulation as a potential therapeutic target in ALI and sepsis-associated inflammation.

## Highlights

• METTL3 promotes NETosis via m6A methylation in neutrophils during ALI.
• SYK is a direct m6A-modified target of METTL3 in neutrophils.
• METTL3 deficiency reduces NET formation and protects against LPS-induced lung injury.
• METTL3 stabilizes SYK mRNA to sustain SYK/ERK/MEK signaling.
• Targeting METTL3 or SYK suppresses NETosis and alleviates pulmonary inflammation.

## Graphical abstract

Schematic model illustrating the role of METTL3-mediated m6A modification in promoting SYK expression, NETosis, and neutrophil-driven lung injury. Pharmacological inhibition of METTL3 suppresses this axis, mitigating inflammation and vascular damage in ALI and sepsis.

**Figure:**
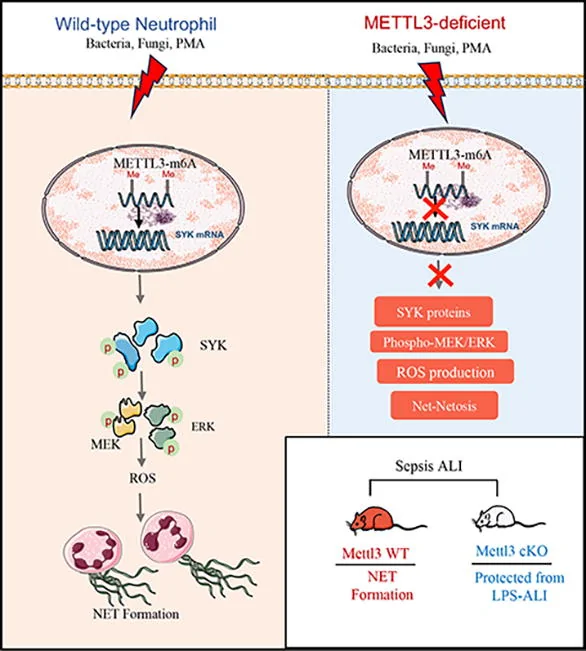

## Introduction

Acute lung injury (ALI) is a severe clinical syndrome characterized by widespread pulmonary inflammation, alveolar epithelial damage, and impaired gas exchange [1], [2], [3], [4]. Despite advances in supportive care, ALI and its more severe form, acute respiratory distress syndrome (ARDS), remain major causes of morbidity and mortality in critically ill patients, particularly in the context of sepsis, trauma, and pneumonia [4], [5], [6], [7]. A hallmark of ALI pathogenesis is the rapid accumulation and activation of neutrophils within the pulmonary microenvironment [8].

Neutrophils serve as the first line of defense against invading pathogens [9], [10], [11], [12]. However, their dysregulated activation can contribute to tissue injury through the release of reactive oxygen species (ROS), proteolytic enzymes, and neutrophil extracellular traps (NETs) [10], [13], [14], [15], [16]. NETs are web-like chromatin structures extruded by neutrophils in response to various stimuli, and while they help to trap and neutralize pathogens, excessive NET formation (NETosis) is increasingly implicated in the progression of ALI [14], [17], [18], [19], [20]. Therefore, understanding the regulatory mechanisms underlying NETosis is crucial for identifying therapeutic strategies to control neutrophil-mediated lung inflammation.

Epitranscriptomic regulation, particularly through N6-methyladenosine (m6A) modification, has emerged as a key modulator of immune cell function [21]. m6A is the most prevalent internal mRNA modification in eukaryotic cells and influences mRNA stability, splicing, export, and translation [21], [22], [23]. The m6A writer enzyme METTL3 catalyzes the deposition of this modification and has been shown to regulate diverse immune processes, including macrophage polarization, T cell homeostasis and neutrophil migration [24], [25], [26], [27]. However, the role of m6A and METTL3 in neutrophil NETosis remains poorly understood.

In this study, we investigated whether METTL3-mediated m6A modification controls NET formation in neutrophils and contributes to lung inflammation during LPS-induced ALI. We identify SYK (Spleen Tyrosine Kinase) as a direct m6A target of METTL3, linking epitranscriptomic regulation to NETosis through the SYK/ERK/MEK signaling axis. Our findings reveal a previously unrecognized mechanism by which METTL3 enhances innate immune responses, providing a foundation for novel m6A-targeted therapies in inflammatory lung diseases.

## Methods

### Mice and treatments

C57BL/6J mice (8–10 weeks old, both sexes) were maintained under specific pathogen-free (SPF) conditions. To generate myeloid- or neutrophil-specific METTL3 knockout mice, METTL3flox/flox mice (kindly provided by Dr. Gaoxiangwei, Zhejiang University) were crossed with Ly6G-Cre or Lyzm-Cre mice (Jackson Laboratory), respectively. Littermates lacking Cre were used as controls.

To induce acute lung injury (ALI), mice were anesthetized and intratracheally administered lipopolysaccharide (LPS, 4 mg/kg body weight, Sigma-Aldrich). For pharmacological inhibition studies, mice received daily intraperitoneal injections of STM2457 (50 mg/kg, Selleckchem) or the SYK inhibitor R406 (10 mg/kg, MedChemExpress) for 2–3 days prior to LPS administration. For NET depletion experiments, mice were treated with DNase I (50 µg, i.p.) immediately after LPS instillation.

### Ethics statement

All experiments involving animals were conducted according to the ethical policies and procedures approved by the ethics committee of the Animal Care and Use Committee of Guangdong Medical University (Approval no. GDY-2202218).

### Neutrophil isolation and cell culture

Mouse bone marrow neutrophils were isolated by flushing femurs and tibias, followed by negative selection using a magnetic separation kit (Miltenyi Biotec). For lung neutrophils, tissues were minced, digested with collagenase I (1 mg/mL), and passed through a 70 µm strainer. Red blood cells were lysed using ammonium chloride buffer.

The human promyelocytic cell line HL-60 (RRID: CVCL_0002. Procell Life Science & Technology Co., Ltd, CL-0110) was cultured in RPMI-1640 medium supplemented with 10% fetal bovine serum (FBS) and 1% penicillin–streptomycin. The HEK293T/17 cells (RRID: CVCL_1926. Procell Life Science & Technology Co., Ltd, CL-0469) was cultured in DMEM medium supplemented with 10% fetal bovine serum (FBS) and 1% penicillin–streptomycin. Those cells were routinely tested for mycoplasma contamination, and all cultures were confirmed to be negative. To differentiate into neutrophil-like cells (dHL60), HL-60 cells were treated with 1.25% dimethyl sulfoxide (DMSO) for 5 days. Neutrophil marker validation (CD11b, MPO) confirming successful differentiation.

### Flow cytometry

Blood or tissue-derived single-cell suspensions were blocked with Fc receptor antibody and stained for 30 min with fluorescent-conjugated antibodies against CD45, Ly6G, CD11b, SYK, TLR2. Neutrophils were identified as Ly6G^+^CD11b^+^ cells. Data acquisition was conducted on a BD LSR II cytometer using FACSDiva software, and results were analyzed with FlowJo.

### Western blot and dot blot

Neutrophils were lysed in RIPA buffer containing protease and phosphatase inhibitors. Protein concentrations were determined by BCA assay, and samples were subjected to SDS-PAGE, transferred to PVDF membranes, and probed with primary antibodies (anti-METTL3, SYK, p-SYK, ERK, p-ERK, MEK, p-MEK, H3Cit, Histone H3, TLR2 and GAPDH).

For dot blot, total RNA was extracted using TRIzol reagent (Invitrogen), denatured at 95°C for 3 min, and spotted onto Amersham Hybond-N + membranes. After UV crosslinking, membranes were blocked and probed with an anti-m6A antibody (Abcam, ab232905).

### Real-time quantitative PCR

RNA was extracted from neutrophils using TRIzol, and cDNA was synthesized using a reverse transcription kit (TaKaRa). Real-time PCR was performed using SYBR Green mix (TaKaRa) on a Roche LightCycler 480. Gene expression was normalized to β-actin or GAPDH using the 2^-ΔCt method.

### Immunofluorescence microscopy

Neutrophils (6 × 10^5) were plated on confocal dishes, allowed to adhere, and stimulated with PMA (100 ng/mL) for 4–6 h, with or without STM2457 or R406. Cells were fixed in 4% paraformaldehyde, permeabilized with 0.5% Triton X-100, and stained with DAPI and Sytox Green or H3Cit antibody according to the manufacturers’ instructions. Images were captured using an Olympus FV3000 confocal microscope. Immunofluorescence images were quantified using ImageJ with uniform thresholds.

### MPO activity assay

MPO activity in lung homogenates or cell pellets was measured using a commercial MPO Activity Assay Kit (Solarbio, BC5715) according to the manufacturer's instructions.

### Evans blue dye assay

Mice were injected intravenously with 20 mg/kg Evans blue dye 30 min before tissue harvest. After euthanasia and PBS perfusion, lungs were excised, weighed, and incubated in formamide at 60°C overnight. Supernatants were collected and absorbance was measured at 620 nm. Evans blue content was normalized to lung weight.

### Histology

Lung tissues were fixed in 4% paraformaldehyde, embedded in paraffin, sectioned at 5 µm, and stained with hematoxylin and eosin (H&E). Slides were imaged under light microscopy. Lung injury was quantified based on four histological features—inflammation, edema, hemorrhage, and alveolar septal thickening—each scored on a 0–4 scale. Inflammation was assessed by counting inflammatory cells per × 100 field in 5–7 random fields. Edema and hemorrhage were graded as 0 (absent), 1 (<10% alveoli), 2 (10–30%), 3 (30–50%), or 4 (>50%). Septal thickening was measured at the thickest point under 400 × magnification and classified as 0 (<15  μm), 1 (15–30  μm), 2 (30–45  μm), 3 (45–60  μm), or 4 (>60  μm). The total lung injury score was calculated as the mean of the four individual scores.

### MeRIP-seq

Total RNA from bone marrow neutrophils was extracted using TRIzol. For MeRIP-seq, RNA was fragmented, immunoprecipitated with anti-m6A antibody, and subjected to high-throughput sequencing (Illumina NovaSeq 6000). Input and m6A-enriched samples were aligned and analyzed for peak calling.

### Sequencing data analysis

Low quality reads were filtered by fastp, Raw reads containing over 50% of low-quality bases or over 10% of N bases were removed. Adapter sequences were trimmed. Reads with length between 20–40 bp were retained for subsequent analysis. The remaining reads were mapped to whole genome alignment by STAR with 2-pass setting enabled. m6A coverages at individual sites were normalized by the mean coverage of the transcript. Differentially expressed genes (DEGs) were identified using DESeq2 (version 1.36.0) in R. Genes with an adjusted p-value (FDR) < 0.05 and |log_2_(fold change)| ≥ 1 were considered significantly differentially expressed. Gene Ontology (GO) pathway enrichment analyses were performed using the cluster Profiler R package. Gene set enrichment analysis (GSEA) was conducted using the fgsea package with MSigDB gene sets.

### MeRIP-qPCR

Enrichment of m6A on specific transcripts was quantified by MeRIP-qPCR using the EpiQuik m6A RNA Enrichment Kit (Epigentek). m6A-bound RNA was isolated, reverse-transcribed, and analyzed via qPCR using specific primers (Table S1).

### mRNA stability and transcription assays

For RNA decay, neutrophils were treated with 5 µM actinomycin D, and RNA was harvested at 0, 1, 2, and 4 h. SYK transcript stability was analyzed by qPCR. For nascent RNA labeling, neutrophils were pulsed with 0.5 mM 5-ethynyl uridine (EU) at different time points. EU-labeled RNA was purified using the Click-iT Nascent RNA Capture Kit (Invitrogen).

### Dual-luciferase reporter assay

Differentially methylated SYK 3′UTR regions between WT and METTL3-KO neutrophils were identified by MeRIP-seq. Wild-type and m6A-mutant 3′ UTRs of SYK were cloned into the GP-miRGLO vector (GenePharma). HEK293T cells (Procell Life Science & Technology Co., Ltd, CL-0469) were co-transfected with these constructs, and luciferase activity was measured 48 h later using the Dual-Luciferase Reporter Assay System (Promega). Firefly luciferase was normalized to Renilla.

### Plasmid construction and mutagenesis

SYK was cloned into the LV5(EF-1a/GFP&Puro) vector at NotI/BamHI sites (GenePharma). Lentiviruses were produced in 293 T cells, filtered (0.45 μm), and injected (40 μL) into the femoral cavity of mice via the kneecap using a 30-gauge insulin syringe. On day 7 after intratracheal LPS challenge, bone marrow and blood cells were analyzed.

### Phagocytosis assay

Neutrophils were incubated with opsonized fluorescent microspheres (Thermo Fisher) at a 10:1 bead-to-cell ratio for 30 min at 37 °C. After washing, cells were analyzed by flow cytometry to quantify bead uptake.

### Mouse MPO-DNA enzyme-linked immunosorbent assay (ELISA)

Mice plasma was collected at indicated time points after treatment. Collect plasma using EDTA or heparin as an anticoagulant. Centrifuge samples for 30 min at 3000 × g at 2-8℃ within 30 min of collection. Mouse MPO-DNA ELISA kits were purchased from MEIMIAN and the ELISA assays were performed according to the manufacturer’s instructions.

### Statistical analysis

All statistical analyses were performed using GraphPad Prism 9. Data are presented as mean ± SEM. Comparisons between two groups were performed using two-tailed unpaired Student’s t-tests unless otherwise indicated. For analyses involving more than two groups, one-way or two-way ANOVA with appropriate post-hoc tests were applied. A p-value < 0.05 was considered statistically significant.

## Results

### METTL3 deficiency alleviates neutrophil recruitment and lung injury in ALI

To explore the role of METTL3 in neutrophils during acute lung injury (ALI), we generated neutrophil-specific METTL3 knockout mice (METTL3flox/flox; Ly6G-Cre, referred to as METTL3 cKO) (Fig. S1A-B). These mice and their littermate controls were subjected to intratracheal lipopolysaccharide (LPS) challenge to induce ALI (Fig. 1A). Six hours after LPS administration, a time point corresponding to peak neutrophil infiltration, lung tissue and bronchoalveolar lavage fluid (BALF) were collected for flow cytometric analysis. We observed a significant reduction in the number of CD45^+^CD11b**^+^**Ly6G**^+^** neutrophils in the lung tissue and BALF of METTL3 cKO mice compared to wild-type (WT) littermates (Fig. 1B-D). Given that neutrophils originate from myeloid cells, we also examined mice with myeloid-specific METTL3 deletion (METTL3flox/flox; Lyzm-Cre) and observed a marked reduction in neutrophil levels (Fig. S1C-D). This result indicates that METTL3 promotes neutrophil recruitment into inflamed lung tissue.

**Fig. 1:**
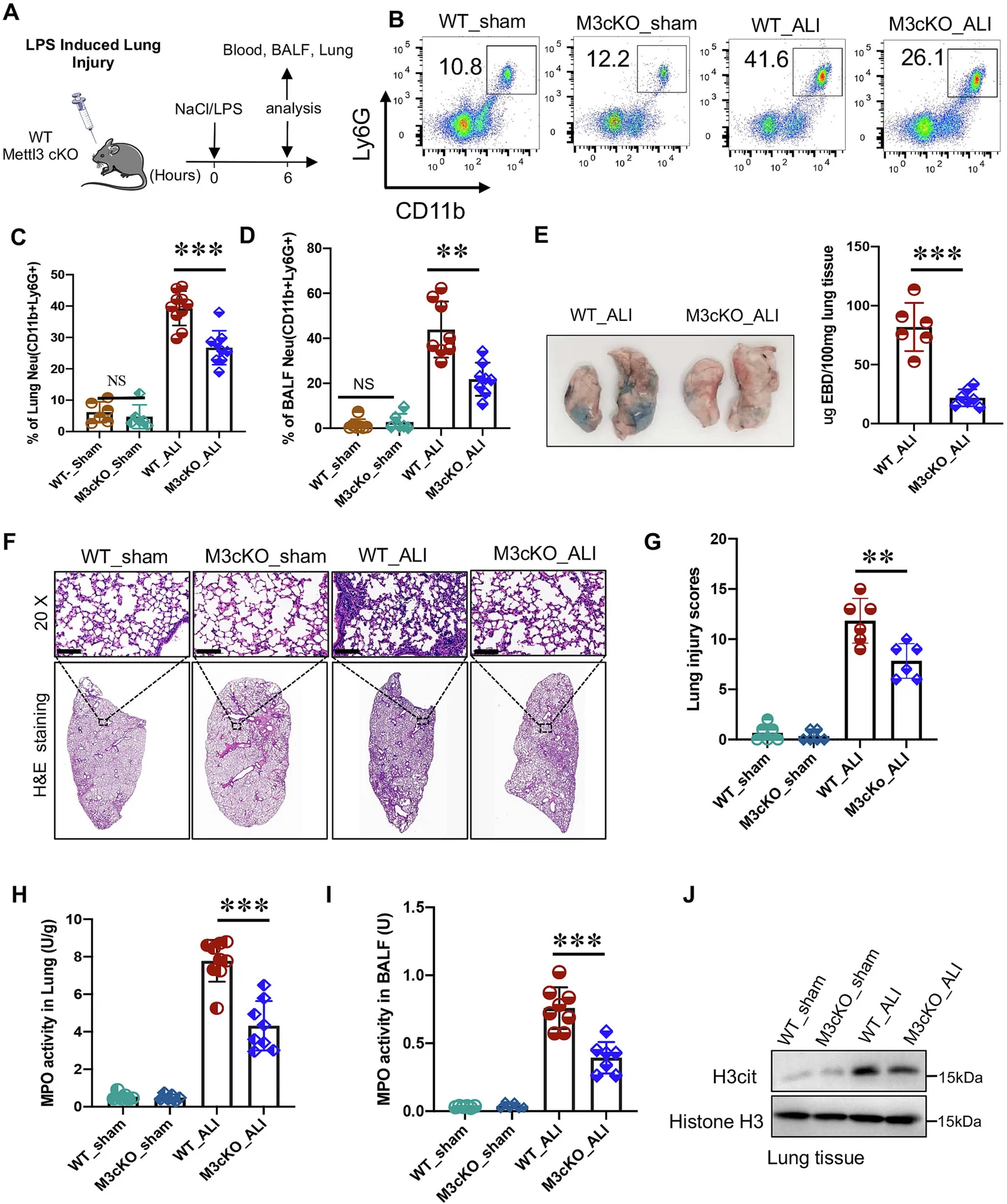
**METTL3 deficiency alleviates neutrophil recruitment and lung injury in ALI.** (A) Schematic of LPS-induced ALI and sample collection. (B–D) Flow cytometry analysis of CD11b + Ly6G + neutrophils (CD45 gated) in lung tissue (B-C) and BALF (D) from WT and METTL3 cKO mice after LPS challenge. (E) Quantification of Evans blue dye extravasation in lungs. (F-G) Representative H&E staining of lung sections and semiquantitative assessments of lung histopathology (scale bar, 100 um). (H–I) MPO activity in lung tissue and BALF cells. (J) Western blot of H3Cit and histone H3 in lung tissue. Data represent mean ± SEM, n ≥ 6 per group. *p < 0.05, **p < 0.01, ***p < 0.001.

Histological examination further confirmed reduced pulmonary inflammation in METTL3 cKO mice. Hematoxylin and eosin (H&E) staining revealed reduced alveolar wall thickening, minimal leukocyte infiltration, and better preservation of alveolar architecture in METTL3 cKO lungs compared to WT controls, which exhibited widespread alveolar collapse, cellular infiltration, and disrupted lung structure (Fig. 1F-G, S1E-F). Importantly, the accumulation of Evans blue in the lung parenchyma was significantly lower in METTL3 cKO mice following LPS challenge, indicating METTL3 driven NET formation contributes to endothelial leakage in ALI (Fig. 1E).

To assess the functional contribution of NETs to lung injury, we treated LPS-challenged WT mice with DNase I, which degrades extracellular DNA in the NETs (Fig. S2A). DNase I treatment did not affect neutrophil recruitment to the lungs during acute lung injury (Fig. S2B-C). However, DNase I treatment significantly improved lung histopathology and endothelial leakage (Fig. 2SD-G), suggesting that NETs play an important role in lung injury during ALI.

To clarify the role of METTL3 in neutrophil NET formation during ALI, markers associated with NET were further analyzed. Myeloperoxidase (MPO) activity—a marker of neutrophil activation—was significantly decreased in whole lung lysates and bronchoalveolar cells from METTL3 cKO mice (Fig. 1H-I, S1G). Histone H3 citrullination (H3Cit), a critical step in chromatin decondensation during NETosis, was also diminished in lung tissues of METTL3-deficient mice, as shown by Western blot (Fig. 1J, S1H).

These collective findings demonstrate that METTL3 is required for efficient neutrophil recruitment and activation during LPS-induced ALI, and its absence confers significant protection against lung inflammation and injury.

### Neutrophil METTL3 promotes NET formation in vitro

To determine whether METTL3 regulates NET formation, we visualized NETs in vitro by staining neutrophils isolated from LPS-treated mice with the membrane-impermeable DNA dye SYTOX Green and the nuclear dye DAPI. Neutrophils from ALI mice exhibited robust NET formation, characterized by extracellular DNA fibers co-stained with both dyes (Fig. 2A-B). Interestingly, m6A RNA modification levels were elevated in blood neutrophils and lung tissue from ALI mice as well as bone marrow derived neutrophils (BMNeu) treated with phorbol 12-myristate 13-acetate (PMA), as detected by dot blot assays (Fig. 2C, S3A-B). This suggests a correlation between NETosis and increased m6A methylation. We examed the main regulators for m6A and found that METTL3 showed significant increase in BMNeu after PMA treatment (Fig. 2D-E, S3C). Similar results were observed in DMSO-differentiated HL60 (dHL60) cells (Fig. S3D-F).

**Fig. 2:**
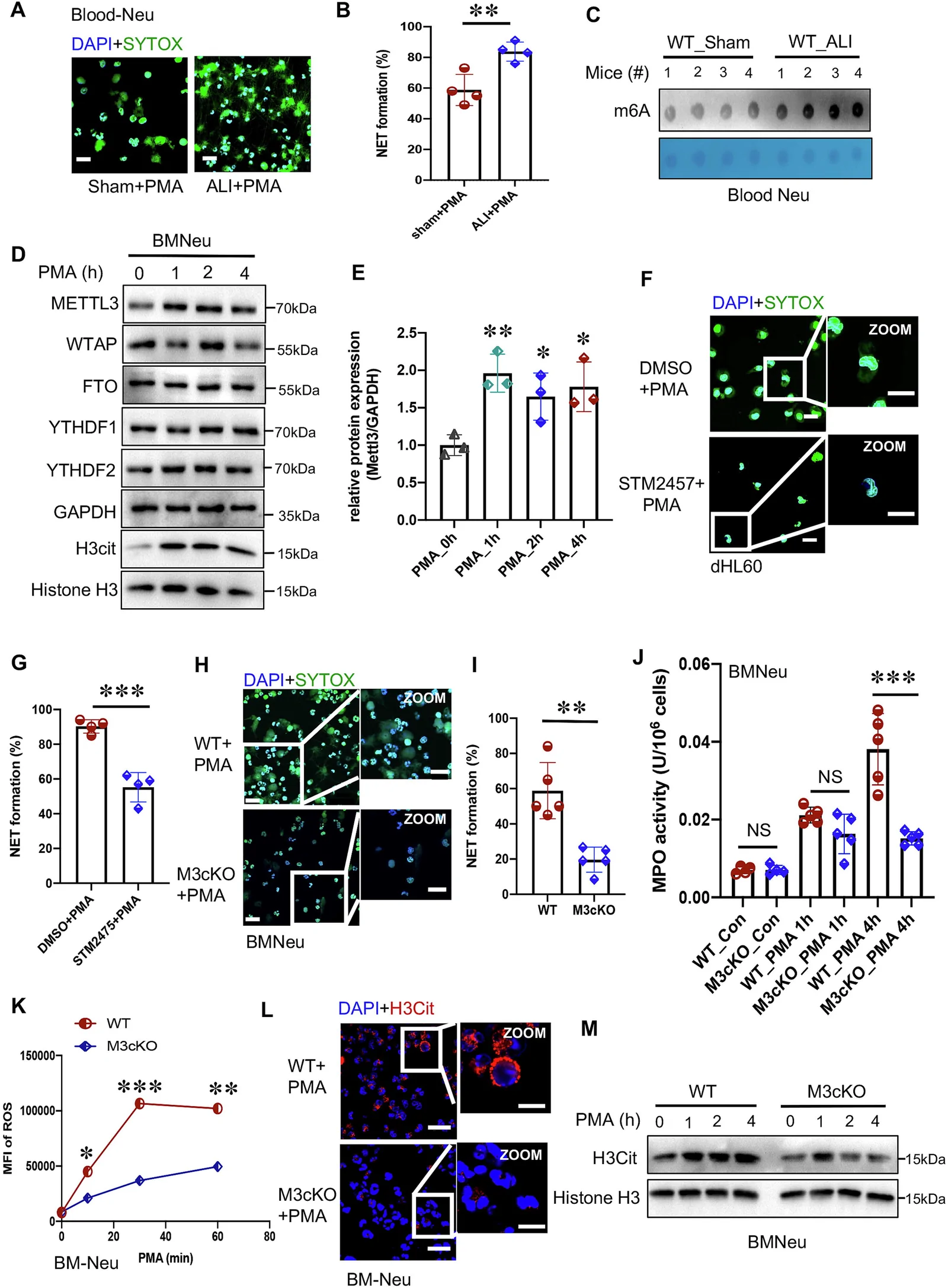
**METTL3 is essential for NET formation.** (A-B) SYTOX Green/DAPI staining of NETs in blood neutrophils (scale bar, 20 um). (C) Dot blot showing m6A levels in blood neutrophils. (D–E) PMA induces METTL3 expression in bone marrow neutrophils. (F–I) Confocal images of SYTOX/DAPI or H3Cit/DAPI staining in STM2457-treated dHL60, PMA-treated WT and METTL3 cKO neutrophils (scale bar, 20 um). (J-M) Quantification of MPO activity, ROS production and H3Cit levels in bone marrow neutrophils (scale bar, 20 um). Data represent mean ± SEM, n ≥ 3 independent experiments. *p < 0.05, **p < 0.01, ***p < 0.001.

To directly assess the function of METTL3 in NET formation, we treated dHL60 cells with the METTL3 inhibitor STM2457. Inhibition of METTL3 reduced NET formation, as evidenced by reduced SYTOX Green-positive extracellular DNA structures (Fig. 2F-G) and global m6A levels (Fig. S3G). To validate these findings genetically, we stimulated bone marrow derived neutrophils (BMNeu) isolated from WT and METTL3 cKO mice with PMA. WT BMNeu treated with phorbol 12-myristate 13-acetate (PMA) showed robust NET formation (Fig. 2H) and membrane rupture regulator NINJ1 mRNA upregulation (Fig. S3H). In contrast, neutrophils from METTL3 cKO mice displayed markedly fewer extracellular DNA structures (Fig. 2H). These cells displayed a ∼ 67% reduction in NET formation compared to WT neutrophils (Fig. 2I). METTL3 deficiency also led to decreased MPO activity (Fig. 2J), ROS production (Fig. 2K) and histone H3 citrullination (Fig. 2L-M, S4A), reinforcing the critical role of METTL3 in the NETosis program. Similar defects were observed in siMETTL3-transfected dHL60 cells (Fig. S3I-K, Fig. S4B). Interestingly, METTL3 loss-of-function in BMNeu and dHL60 also impaired phagocytosis of E. coli (Fig. S4C, D).

Taken together, these data demonstrate that METTL3 promotes NET formation and triggers downstream effector functions.

### METTL3 promotes NETosis via SYK/ERK/MEK signaling axis

Dot blot analysis confirmed that METTL3 deficiency results in decreased m6A modification in neutrophils (Fig. 3A). To delineate how METTL3 regulates NETosis, we performed MeRIP-seq. Differential m6A peak enrichment highlighted significant changes in the ERK/MAPK pathway and ROS production, both essential for NET formation (Fig. 3B). We next performed Venn diagram-based enrichment analysis to identify potential target genes, which revealed three candidates: SYK, TLR4, and Ptk2b (Fig. 3C). qPCR confirmed that only SYK, a non-receptor tyrosine kinase required for neutrophil activation, was significantly downregulated in METTL3-deficient (M3cKO) neutrophils (Fig. 3D). SYK mRNA and protein expression dramatically decreased in METTL3 cKO BMNeu and METTL3 KD dHL60 at baseline as well as after PMA treatment (Fig. 3E-G, S5A-B).

**Fig. 3:**
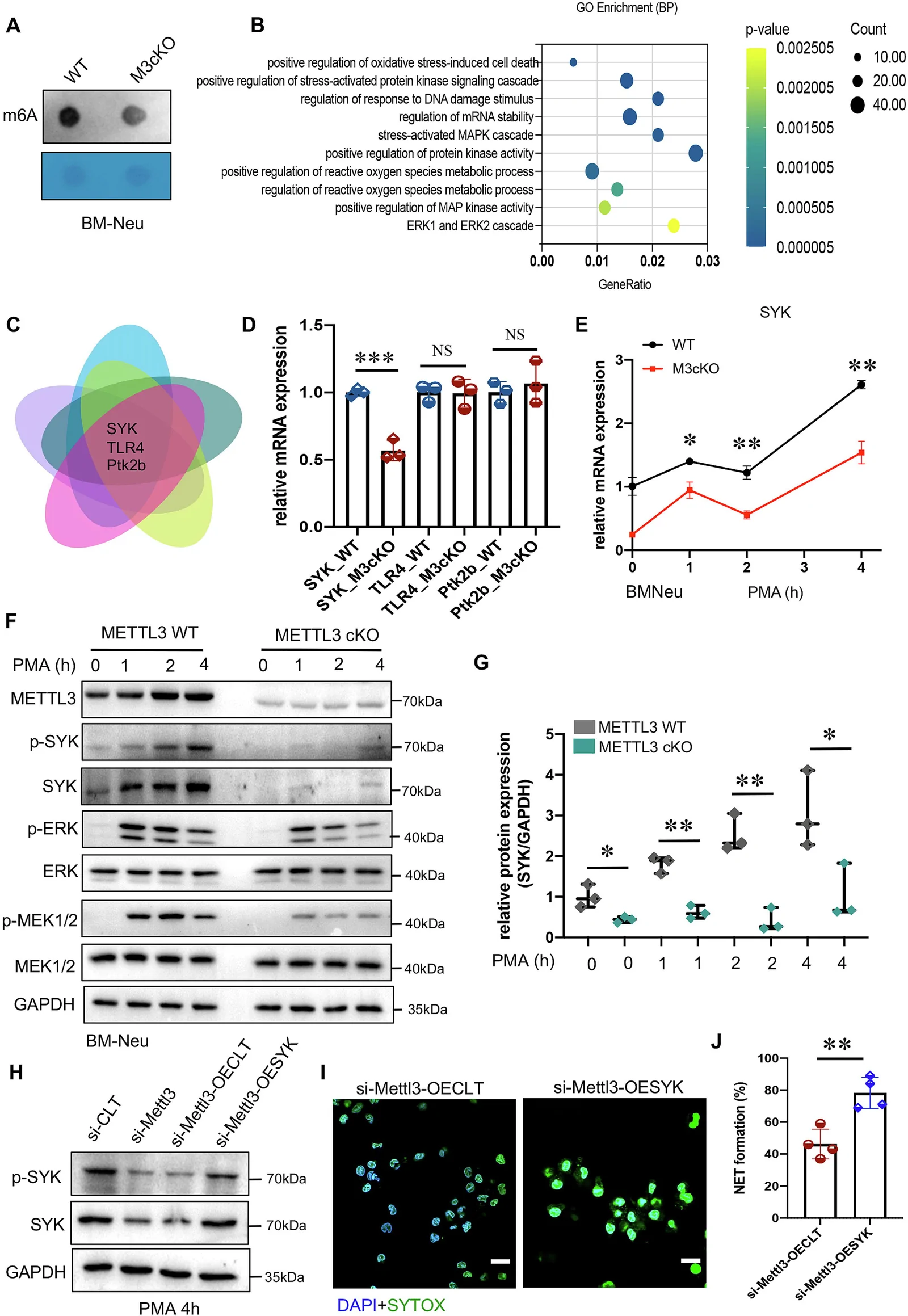
**METTL3 promotes NETosis via stabilization of SYK mRNA.** (A) Dot blot of m6A levels in WT and METTL3 KO neutrophils. (B) GO enrichment analysis of MeRIP-seq data in the biological process category of transcripts bearing m6A modification in WT and METTL3 KO BM neutrophils. (C) Venn diagram illustrating the overlap of enriched genes in Net formation related GO pathway enrichment analysis of MeRIP-seq data. (D) mRNA levels of SYK, TLR4 and Ptk2b in bone marrow neutrophils. (E) SYK mRNA levels in WT and METTL3-deficient neutrophils upon PMA stimulation. (F-G) Western blots of SYK, ERK, and MEK phosphorylation in response to PMA. (H) SYK and p-SYK expression in siMETTL3 dHL60 cells after SYK overexpression in response to PMA. (I–J) SYK overexpression increase NET formation in siMETTL3 dHL60 cells (scale bar, 20 um). Data are from ≥ 3 biological replicates. *p < 0.05, **p < 0.01, ***p < 0.001.

To confirm the importance of SYK in NETosis, we pretreated BMNeu and dHL60 with the SYK phosphorylation inhibitor R406. This treatment significantly reduced NET formation in both primary bone marrow neutrophils and dHL60 cells, as shown by reduced SYTOX staining and decreased H3Cit levels (Fig. S6A-D).

Moreover, ERK/MEK activation was confirmed to be substantially reduced in METTL3-deficient (M3cKO) neutrophils and METTL3 KD dHL60 compared to controls during NETosis (Fig. 3F, S5B). Interestingly, METTL3 inhibitor STM2457 suppressed activation of SYK, ERK, and MEK, key signaling components in NETosis (Fig. S5C), while SYK inhibitor R406 suppressed activation of ERK, and MEK (Fig. S6E-F). Importantly, overexpression of SYK in siMETTL3 dHL60 cells (Fig. S5D-E) increased the levels of PMA-induced SYK phosphorylation (Fig. 3H) and restored NET formation following PMA stimulation (Fig. 3I-J).

These data confirmed that SYK is a direct m6A target of METTL3, and regulates NETosis through the SYK/ERK/MEK signaling axis.

### METTL3-dependent SYK expression promotes sepsis-induced lung injury

We next explored the in vivo relevance of the METTL3-SYK axis in models of ALI. Flow cytometric analysis revealed higher SYK expression in lung-infiltrating neutrophils from WT mice compared to METTL3 cKO mice following LPS challenge (Fig. 4A-B, S7A-B). Similar trends were observed in blood and bone marrow neutrophils of mice subjected to cecal ligation and puncture (CLP) (Fig. S7C-F), a clinically relevant sepsis model.

Next, we overexpressed SYK via intrafemoral lentiviral injection in METTL3 cKO mice. The success of SYK overexpression in lung tissue and neutrophil was confirmed by flow cytometry staining as well as Western Blot analysis (Fig. 4C-D, Fig. S7G-J). SYK overexpression abrogated the beneficial effects in METTL3 cKO ALI (Fig. 4E-F). Importantly, SYK overexpression restored H3Cit levels in lung tissue, indicating that METTL3-driven SYK expression promotes lung injury progression primarily via NET formation (Fig. 4G).

**Fig. 4:**
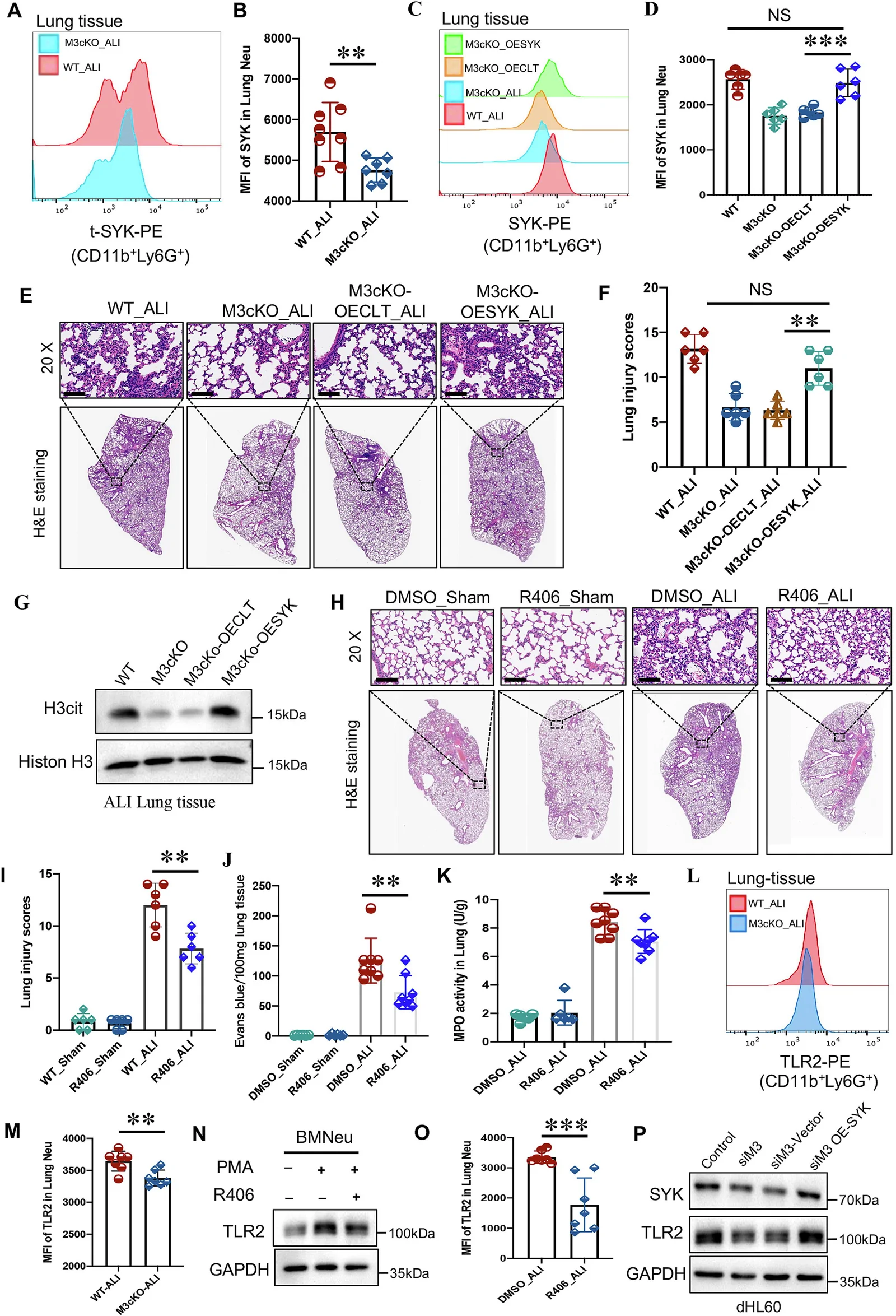
**METTL3-dependent SYK expression contributes to sepsis-induced lung injury** (A–B) Flow cytometry of SYK in lung neutrophils of LPS- induced ALI mice. (C-D) SYK expression after SYK overexpression in vivo. (E-F) Lung histology and semiquantitative histological scores of lung injury after SYK overexpression in vivo (scale bar, 100 um). (G) H3Cit levels after SYK overexpression in vivo. (H-I) Lung histology and semiquantitative assessments of lung histopathology with R406 or vehicle (scale bar, 100 um). (J) Evans blue dye extravasation with R406 or vehicle. (K) R406 reduces MPO activity in lung tissue. (L–M) TLR2 surface expression in WT and METTL3-deficient neutrophils. (N–O) SYK inhibition downregulates TLR2 expression in neutrophils. (P) SYK overexpression restores TLR2 expression. Data represent mean ± SEM, n ≥ 6 per group. *p < 0.05, **p < 0.01, ***p < 0.001.

To assess the role of SYK in sepsis-induced lung injury, mice were pretreated with the SYK phosphorylation inhibitor R406 via intraperitoneal injection for two days prior to ALI induction. R406 treatment alleviated lung injury, as shown by histopathology and Evans blue assays, and reduced MPO activity and H3Cit in the lungs (Fig. 4H-K). While total SYK expression remained unchanged (Fig. S8A-B). R406 effectively suppressed SYK phosphorylation in lung tissues (Fig. S8C).

We also noted a decrease in membrane-bound TLR2 expression on METTL3-deficient neutrophils in ALI lungs (Fig. 4L-M) and in CLP models (Fig. S8D-G). To determine whether TLR2 is a direct m6A target, we performed MeRIP-seq and MeRIP-qPCR, which identified m6A enrichment in the 5′ UTR of TLR2 mRNA (Fig. S9A-B). However, mRNA decay assays showed no difference in TLR2 stability between WT and METTL3-deficient cells (Fig. S9C), suggesting indirect regulation.

Mechanistically, SYK appears to regulate TLR2 expression. Intraperitoneal administration of R406 effectively reduced TLR2 expression in neutrophil during acute lung injury (Fig. 4N-O, S9D-F), while SYK overexpression restored TLR2 expression in METTL3-deficient cells (Fig. 4P). These data suggest that METTL3 regulates TLR2 indirectly through its effects on SYK, linking METTL3 activity to innate immune receptor expression and function.

These findings identify SYK as a key downstream effector of METTL3 in sepsis-induced ALI and a direct downstream effector of METTL3 in the regulation of NETosis.

### METTL3 stabilizes SYK mRNA via m6A modification

To further investigate the mechanism by which METTL3 controls SYK expression, we analyzed m6A modifications on SYK transcripts using MeRIP-seq. We identified strong m6A peaks in the 3′ UTR of SYK mRNA in WT neutrophils, which were significantly reduced in METTL3-deficient cells (Fig. 5A). MeRIP-qPCR confirmed that SYK transcripts were preferentially methylated in WT cells (Fig. 5B).

**Fig. 5:**
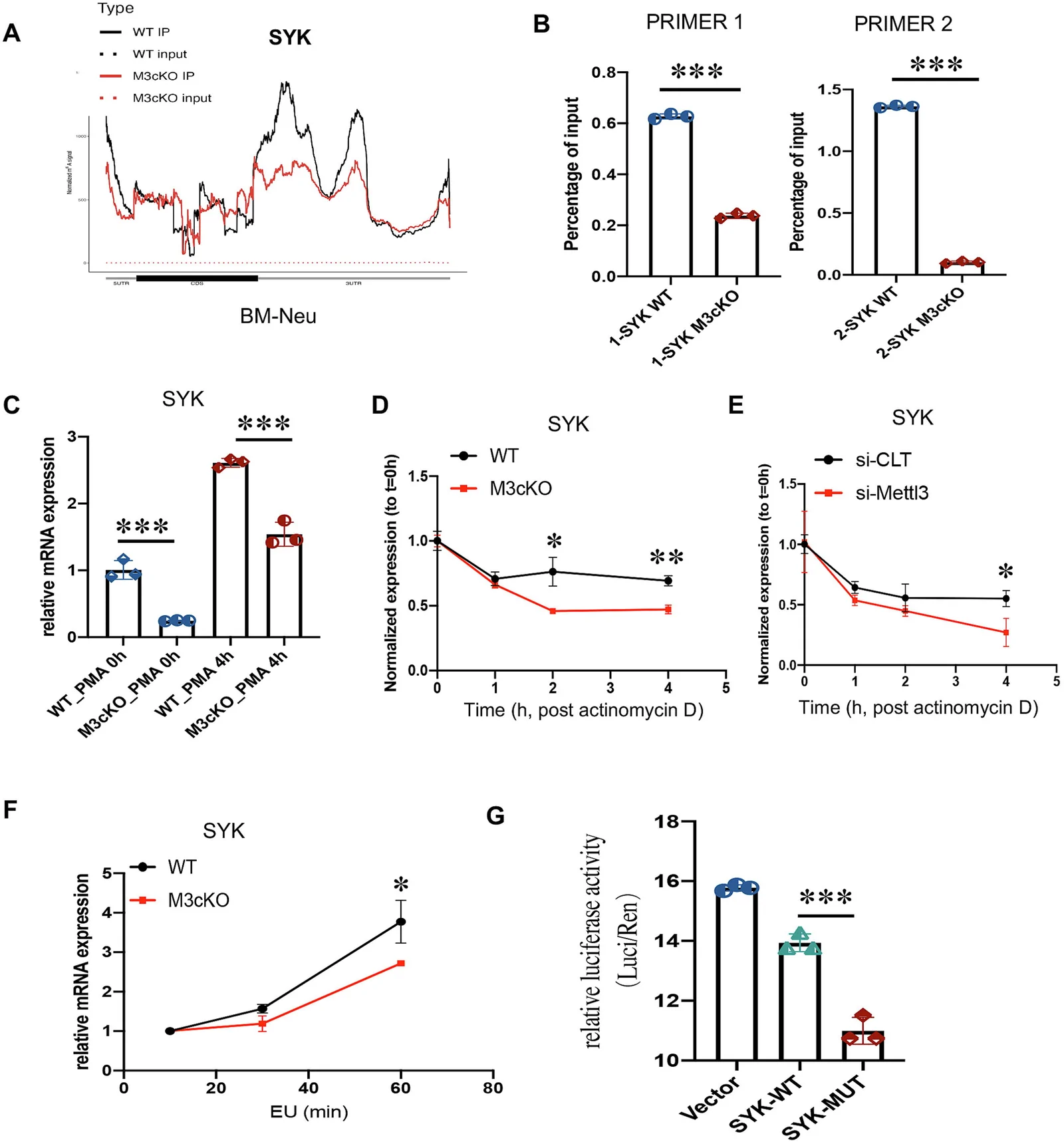
**METTL3 stabilizes SYK mRNA via m6A modification**. (A) MeRIP-seq tracks showing m6A peaks in SYK 3′ UTR from WT but not METTL3 cKO neutrophils. (B–C) MeRIP-qPCR and qPCR of SYK transcripts. (D–F) Actinomycin D and EU pulse-chase show METTL3 prolongs SYK mRNA stability. (G) Luciferase reporter assays using WT and m6A-mutant SYK 3′ UTRs. Data represent mean ± SEM, n = 3 per group. *p < 0.05, **p < 0.01, ***p < 0.001.

Quantitative PCR revealed lower SYK mRNA levels in METTL3-deficient neutrophils upon PMA stimulation (Fig. 5C). To distinguish between transcriptional and post-transcriptional regulation, we performed actinomycin D chase experiments. SYK mRNA degraded more rapidly in METTL3-deficient cells, indicating that METTL3 prolongs SYK transcript stability (Fig. 5D-E). By contrast, nascent RNA labeling showed only a modest reduction in SYK transcription (Fig. 5F).

Luciferase reporter assays using constructs with WT or mutant SYK 3′ UTRs demonstrated that loss of m6A sites significantly reduced reporter activity (Fig. 5G). These data collectively demonstrate that METTL3-mediated m6A modification stabilizes SYK mRNA, supporting sustained protein expression and downstream signaling essential for NET formation.

### Pharmacological inhibition of METTL3 suppresses NETosis and improves lung outcomes

To evaluate the therapeutic potential of targeting METTL3, we pretreated WT mice with METTL3 inhibitor STM2457 [28] before LPS challenge. STM2457 treatment significantly reduced m6A level in lung tissue and sorted blood neutrophils (Fig. 6A, S10A). Importantly, neutrophil accumulation in lung tissue, as measured by flow cytometry, significantly decreased in STM2457 treated mice (Fig. 6B-C). Histological analysis revealed improved lung morphology in STM2457-treated mice, with less alveolar collapse and inflammatory infiltration (Fig. 6D-E). Lung vascular leakage, quantified by Evans blue dye, was also markedly decreased (Fig. 6F, S10B). Flow cytometry showed that STM2457 lowered both SYK expression levels and the percentage of SYK-positive neutrophils in lung tissue (Fig. 6G-I, S10C). Biochemical analysis confirmed reduced MPO activity and H3Cit expression in lung tissue following treatment (Fig. 6J-K), suggesting that inhibition of METTL3 disrupts key signaling pathways involved in NETosis and inflammation in ALI.

**Fig. 6:**
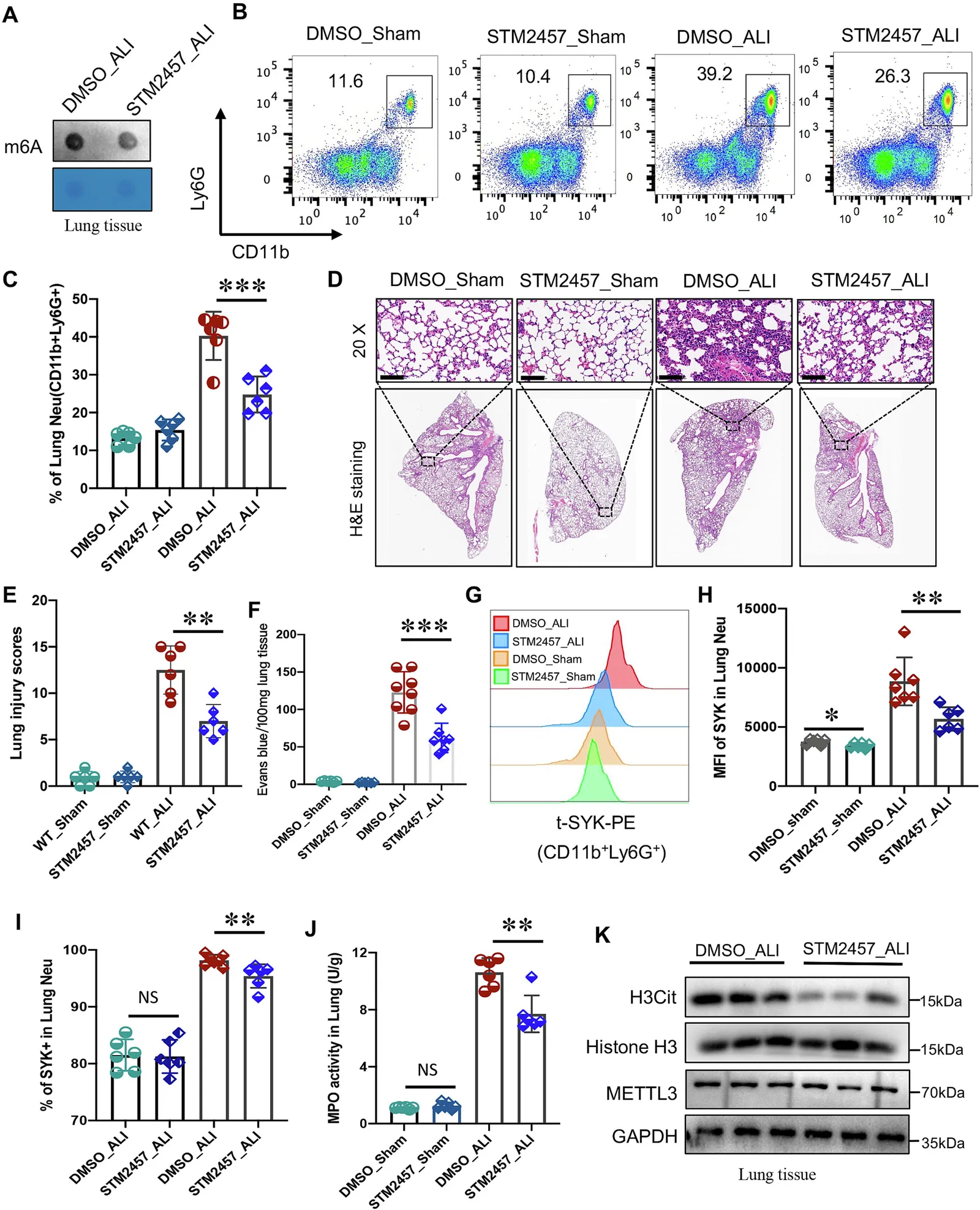
Pharmacological inhibition of METTL3 suppresses NETosis and lung inflammation. (A) STM2457 reduces m6A level in lung tissue. (B–C) STM2457 reduces neutrophil (CD45 gated) infiltration in LPS-induced ALI. (D-E) Lung histology and semiquantitative assessments of lung histopathology with STM2457 or vehicle (scale bar, 100 um). (F) Evans blue quantification. (G–H) Flow cytometry of SYK in lung neutrophils after STM2457 treatment. (I) STM2457 reduces SYK positive neutrophil infiltration in LPS-induced ALI. (J) Quantitative analysis of MPO activity. (K) Western blots showing reductions in H3Cit. Data represent mean ± SEM, n ≥ 6 per group. *p < 0.05, **p < 0.01, ***p < 0.001.

These findings demonstrate that pharmacologic inhibition of METTL3 attenuates NET formation, neutrophil activation, and pulmonary injury, and may represent a promising strategy for the treatment of ALI and sepsis.

Overall, our results uncover a critical epigenetic mechanism by which METTL3-mediated m6A modification of SYK mRNA enhances its stability and promotes NET formation via the SYK/ERK/MEK pathway. This cascade contributes to neutrophil-driven inflammation and lung injury in sepsis and ALI. Both genetic ablation and pharmacological inhibition of METTL3 significantly reduced NETosis, preserved lung tissue integrity, and improved vascular function. Our results highlight METTL3 as a potential therapeutic target for treating inflammatory lung diseases characterized by excessive neutrophil activation.

## Discussion

Neutrophils are critical for host defense, but their dysregulated activation contributes to tissue injury in a variety of inflammatory conditions, including acute lung injury (ALI) and sepsis[9], [10], [16]. One key mechanism by which neutrophils inflict damage is through the release of neutrophil extracellular traps (NETs), a process known as NETosis. While NETs are essential for trapping and neutralizing pathogens, excessive NET formation exacerbates inflammation, disrupts vascular integrity, and promotes organ dysfunction[14], [15], [29], [30]. Previous work[31] demonstrated that METTL3 regulates neutrophil mobilization from the bone marrow through TLR4-dependent signaling in sepsis. Consistently, in the present study, we further confirmed that METTL3 deficiency significantly reduces neutrophil recruitment in LPS-induced ALI. Importantly, our study reveals a previously unrecognized role for the m6A methyltransferase METTL3 in regulating neutrophil extracellular trap (NET) formation and the pathogenesis of acute lung injury (ALI). We demonstrate that METTL3 is essential for sustaining neutrophil effector functions during inflammation by stabilizing Syk mRNA through m6A modification, thereby promoting activation of the SYK/ERK/MEK signaling pathway critical for NETosis.

NETosis is a specialized form of neutrophil activation that involves chromatin decondensation and extracellular DNA release[14], [15]. While several transcriptional and post-translational events have been implicated in NET formation, epitranscriptomic regulation remains poorly defined. Our data provide direct evidence that m6A methylation, catalyzed by METTL3, is indispensable for efficient NET generation both in vivo and in vitro.

We show that conditional deletion of METTL3 in myeloid or neutrophil compartments protects against LPS-induced lung inflammation by reducing neutrophil infiltration, tissue damage, and NET deposition. These findings are in line with previous studies linking excessive NET formation to the exacerbation of ALI and acute respiratory distress syndrome (ARDS)[8], [17], [32]. Importantly, pharmacological inhibition of METTL3 with STM2457 phenocopied the genetic knockout and suppressed NET formation. This convergence highlights METTL3 as a clinically relevant target and supports METTL3 inhibition as a promising therapeutic strategy for NET-driven acute lung injury (ALI).

Mechanistically, we identify Spleen Tyrosine Kinase (SYK) as a direct downstream effector of METTL3. Growing evidence indicates that SYK functions as a central hub integrating multiple NET-inducing pathways, including PMA–PKC, LPS–TLR4/ERK, FcγR, and β2-integrin signaling[33], [34], [35]. Through this convergence, SYK critically drives the MEK/ERK–ROS axis, promoting chromatin remodeling and NET formation. m6A-RIP and RNA decay assays confirm that METTL3 directly methylates and stabilizes Syk mRNA, allowing for sustained SYK protein expression and downstream activation of ERK and MEK signaling. This cascade is well-established in NETosis and is further validated in our model, as SYK inhibition suppresses ERK/MEK activation and impairs NET formation.

Crucially, SYK overexpression in METTL3-deficient neutrophils restores ERK/MEK phosphorylation and rescues the defective NET phenotype, establishing a causal link between METTL3-mediated m6A modification and neutrophil function. Our data further show that METTL3 controls this SYK-centered axis, thereby regulating NETosis irrespective of the initiating stimulus. These findings extend the role of METTL3 beyond its known functions in macrophage polarization and lymphocyte biology, highlighting its specific relevance in neutrophil-mediated inflammation.

In addition to its impact on NETosis, METTL3 deficiency also compromises neutrophil ROS production and bacterial killing, indicating broader impairment of antimicrobial immunity. Given the dual role of NETs in host defense and tissue injury, targeting METTL3 may offer a strategy to mitigate inflammatory damage without fully abolishing innate immune defense.

In conclusion, our study uncovers an epitranscriptomic mechanism whereby METTL3 regulates neutrophil activation and NETosis via the SYK/ERK/MEK axis. These findings suggest that m6A-dependent RNA regulation is a critical layer of immune control and offer a new therapeutic avenue for the treatment of ALI and other neutrophil-driven diseases.

## Study approval

All experiments involving animals were conducted according to the ethical policies and procedures approved by the ethics committee of the Animal Care and Use Committee of Guangdong Medical University (Approval no. GDY-2202218).

## Data availability

All data generated or analyzed during this study are included in this published article (and its supplementary information files). MeRIP-seq data is available in the Gene Expression Omnibus database (https://www.ncbi.nlm.nih.gov/geo/) under accession number GSE225143.

## Author contributions

SL and QZ designed and performed the majority of the experiments. SH, QZ and WZ performed western blotting, flow cytometry and animal experiments. LZ and CL contributed to cells cultured and immunofluorescence. JT, JX and SL performed data analysis and wrote the manuscript. JT, LZ and FX assisted with data interpretation and manuscript writing. JX and JT conceived and supervised the study.

## Compliance with Ethics Requirements

C57BL/6J mice (8-10 weeks old, both sexes) were maintained under specific pathogen-free (SPF) conditions. To generate neutrophil- or myeloid-specific METTL3 knockout mice, METTL3flox/flox mice (kindly provided by Dr. Gaoxiangwei, Zhejiang University) were crossed with Ly6G-Cre or Lyzm-Cre mice (Jackson Laboratory), respectively. Littermates lacking Cre were used as controls. All experiments involving animals were conducted according to the ethical policies and procedures approved by the ethics committee of the Animal Care and Use Committee of Guangdong Medical University (Approval no. GDY-2202218).

## Declaration of competing interest

The authors declare that they have no known competing financial interests or personal relationships that could have appeared to influence the work reported in this paper.
