# Supplementary material for: METTL3 promotes neutrophil extracellular trap formation via SYK/ERK/MEK signaling during acute lung injury
Source: J Adv Res. 2026 Jan 22;88:367–79. doi: 10.1016/j.jare.2026.01.044 (PMC13669593; doi:10.1016/j.jare.2026.01.044)
Supplement: Supplementary file 1 [file mmc1.pdf]

Supplemental Figures

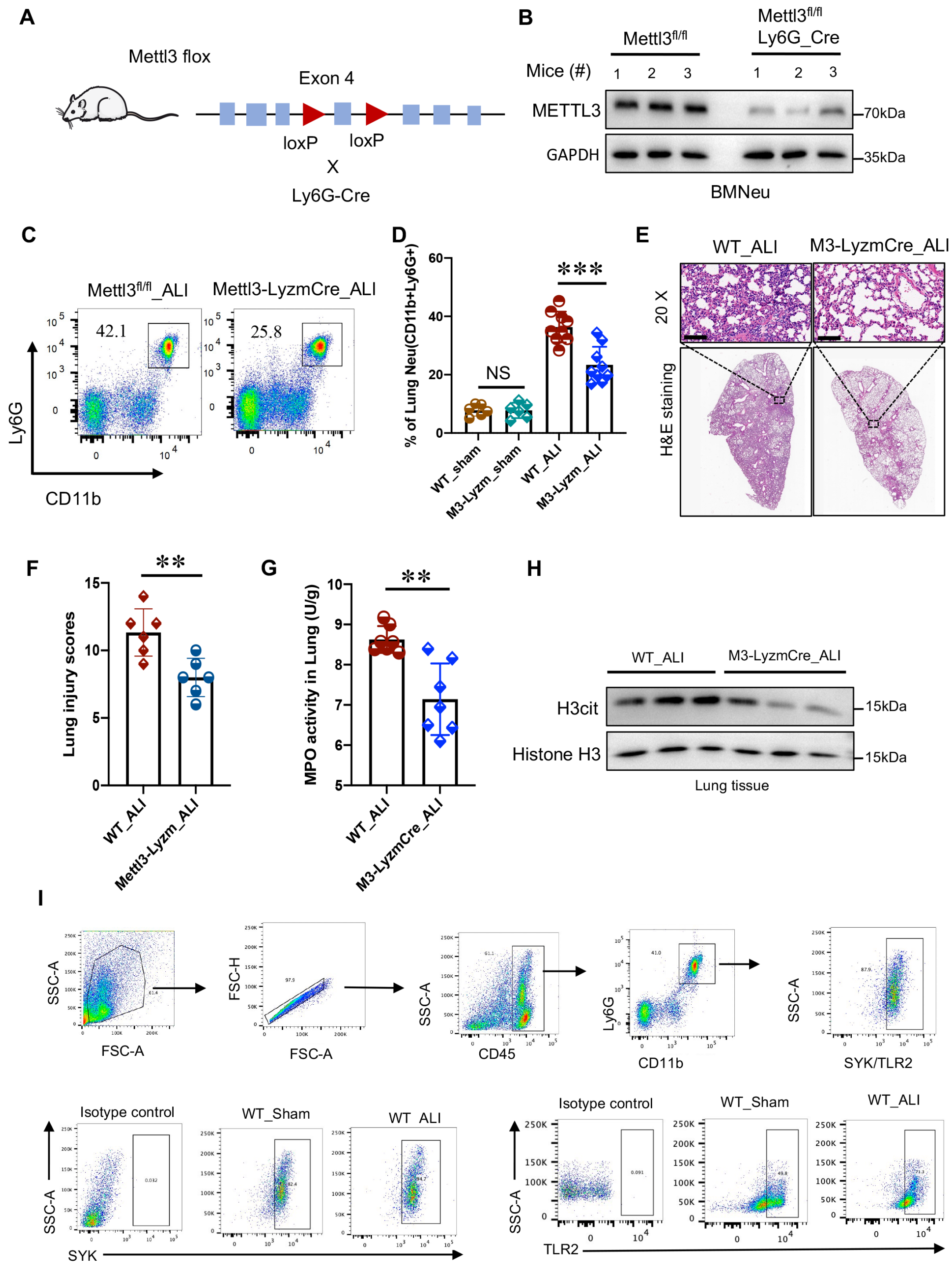

Supplemental Fig.1. METTL3 deficiency alleviates neutrophil recruitment and lung injury in ALI

(A) LoxP sites were inserted into the first and last introns flanking Exon 4 using

CRISPR/Cas9. (B) Western blots show that Mettl3 level of neutrophils in Mettl3f/f Ly6G-Cre mice was effectively reduced. (C-D) Flow cytometry analysis of CD11b+Ly6G+ neutrophils (CD45 gated) in lung tissue of Mettl3f/f Lyzm-Cre mice and Mettl3f/f littermates. (E-F) Representative H&E staining of lung sections and semiquantitative assessments of lung histopathology (scale bar, 100 um). (G) Quantification of MPO activity in lung tissue. (H) Quantification of H3Cit levels in lung tissue. (I) Schematic diagram of the flow cytometry gating strategy. Data represent mean $\pm$ SEM, $n \geq 6$ per group. *$p < 0.05$, **$p < 0.01$, ***$p < 0.001$.

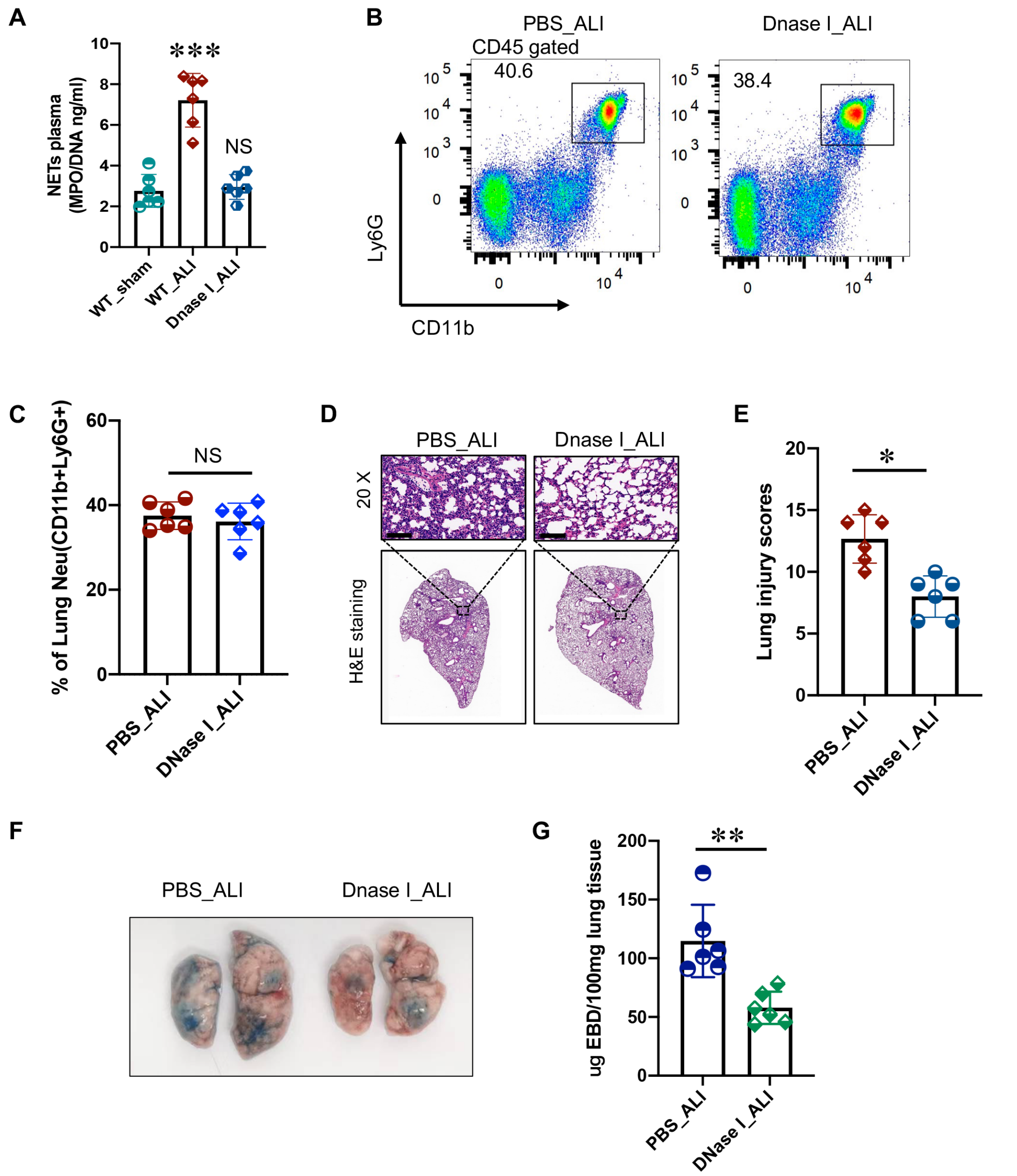

Supplemental Fig.2. Dnase I reduces lung injury in ALI

(A) The concentrations of MPO/DNA NETs in the plasma of ALI mice. (B-C) Neutrophil populations (CD45 gated) in lung tissue from C57BL/6J WT mice treated with or without DNase I. (D-E) H&E staining of lung sections and semiquantitative assessments of lung histopathology (scale bar, 100 um). (F-G) DNase I treatment reduces lung vascular leakage in ALI. Data represent mean ± SEM, n ≥ 6 per group. *p

< 0.05, **p < 0.01, ***p < 0.001.

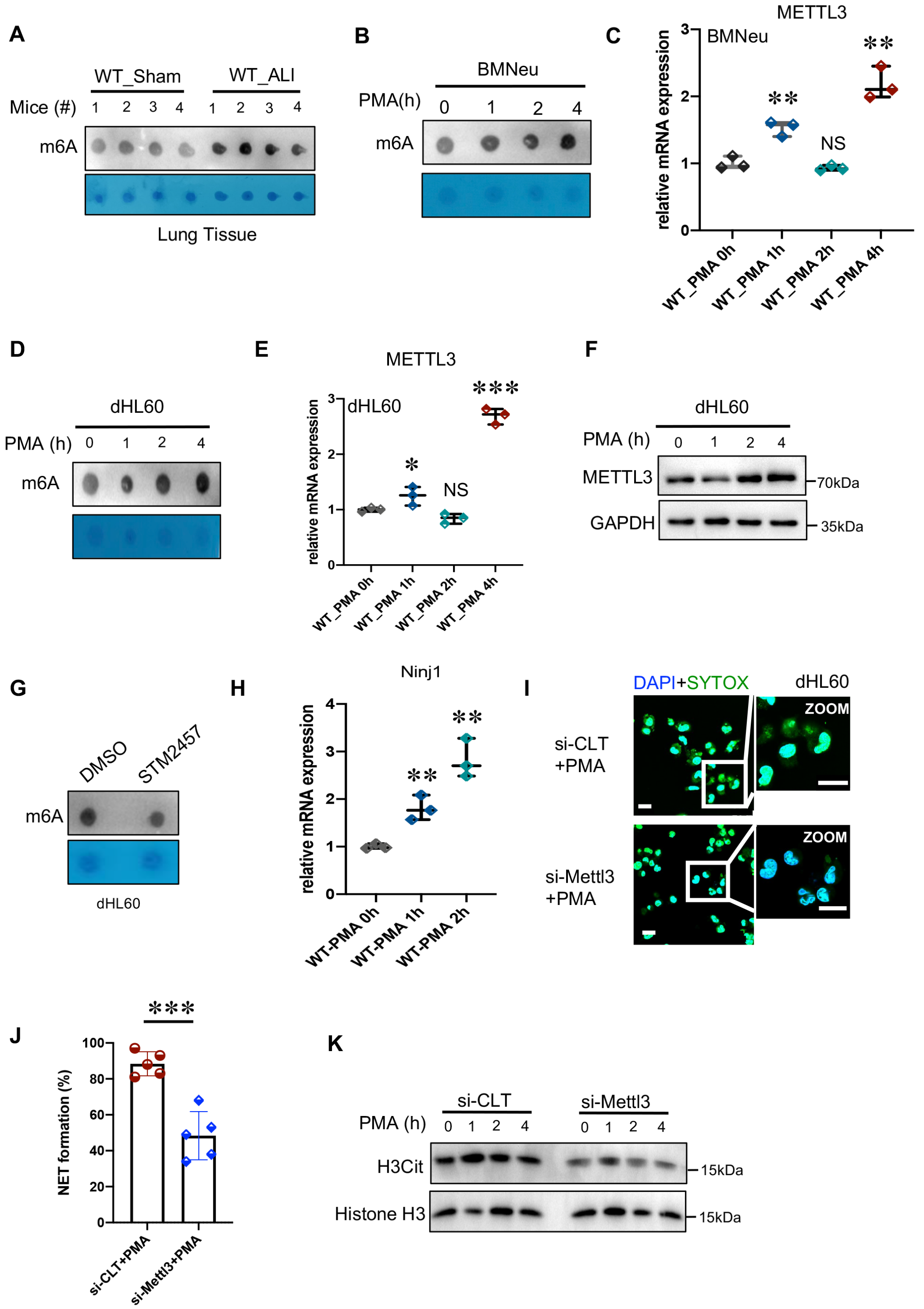

Supplemental Fig. 3. METTL3 promotes NET formation.

(A) Dot blot showing m6A levels in lung tissue. (B–F) PMA induces m6A and METTL3 expression in WT bone marrow and dHL60 neutrophils in response to PMA.

(G) Dot blot showing m6A levels in dHL60 neutrophils with STM2457 treatment. (H) Ninj1 mRNA levels in PMA-treated neutrophils. (I-J) SYTOX Green/DAPI staining of NETs in dHL60 neutrophils (scale bar, 20 um). (K) Quantification of H3Cit levels in dHL60 neutrophils. Data represent mean ± SEM, n ≥ 3 per group. *$p < 0.05$, **$p < 0.01$, ***$p < 0.001$.

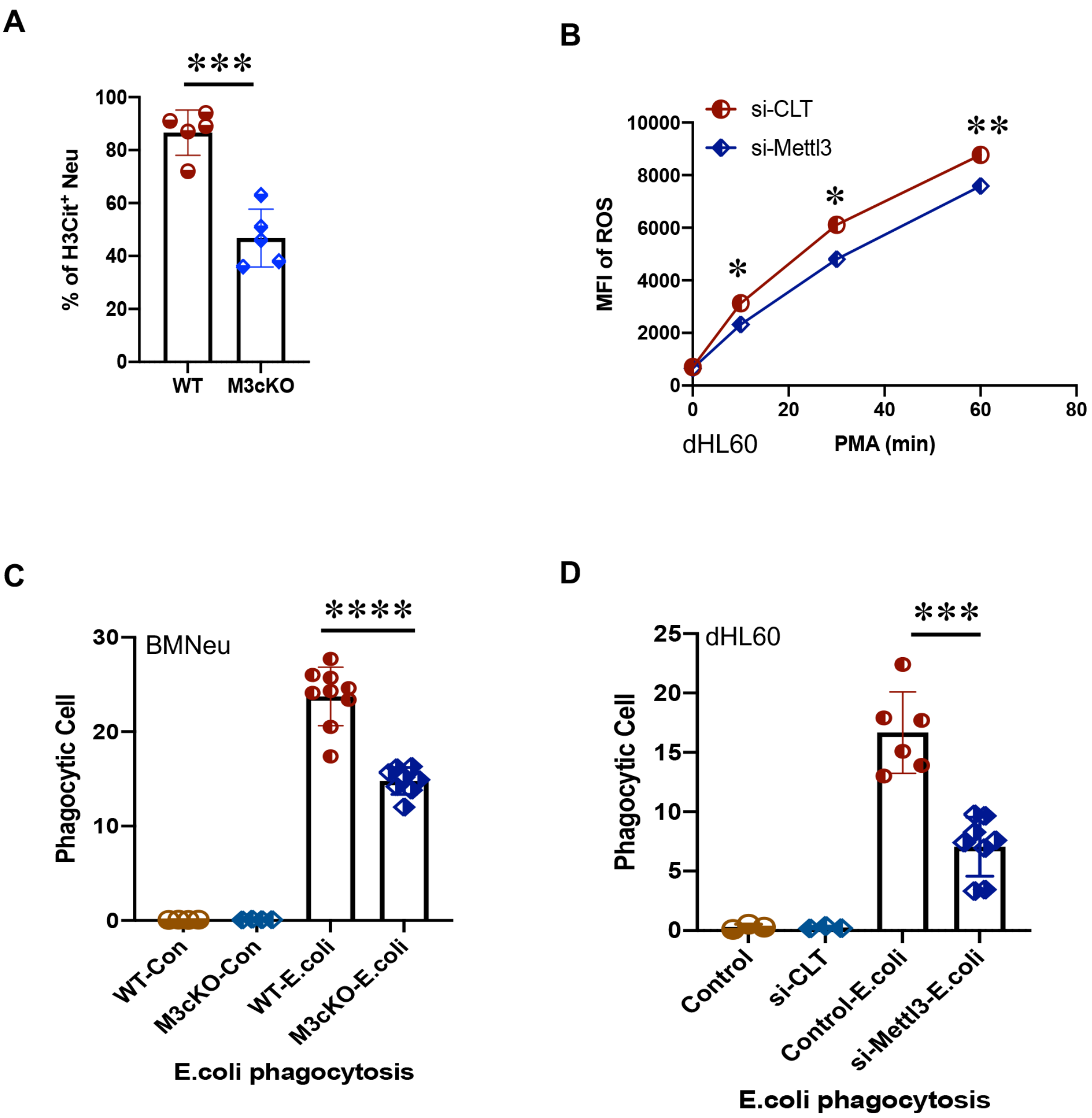

Supplemental Fig. 4. METTL3 promotes neutrophil ROS production and Phagocytosis.

(A) Quantification of H3Cit+ bone marrow neutrophils. (B) Quantification of ROS generation in dHL60 neutrophils. (C-D) Quantification of phagocytosis ability in bone marrow neutrophils and dHL60 neutrophils. Data represent mean ± SEM, $n \geq 3$ per group. *$p < 0.05$, **$p < 0.01$, ***$p < 0.001$.

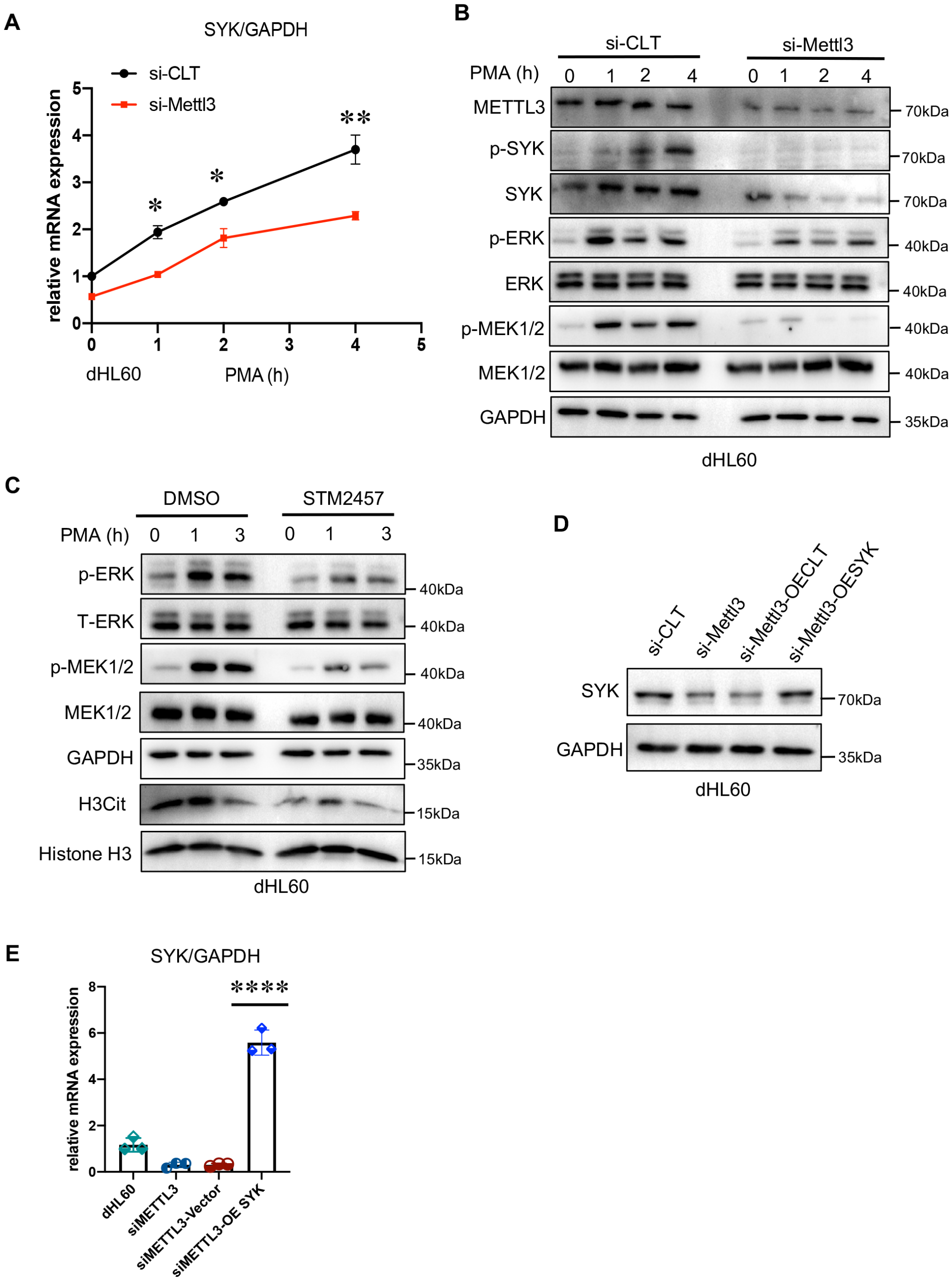

Supplemental Fig. 5. METTL3 inhibition reduces neutrophil ERK/MEK pathway activation.

(A) SYK mRNA levels in dHL60 neutrophils upon PMA stimulation. (B) Western blots of SYK, ERK, and MEK phosphorylation in response to PMA in dHL60 neutrophil. (C) Western blots of ERK, and MEK phosphorylation in response to PMA in dHL60

neutrophil with STM2457 or vehicle. (D-E) SYK protein and mRNA expression in siMettl3 dHL60 cells after SYK overexpression. Data represent mean ± SEM, n ≥ 3 per group. *$p < 0.05$, **$p < 0.01$, ***$p < 0.001$.

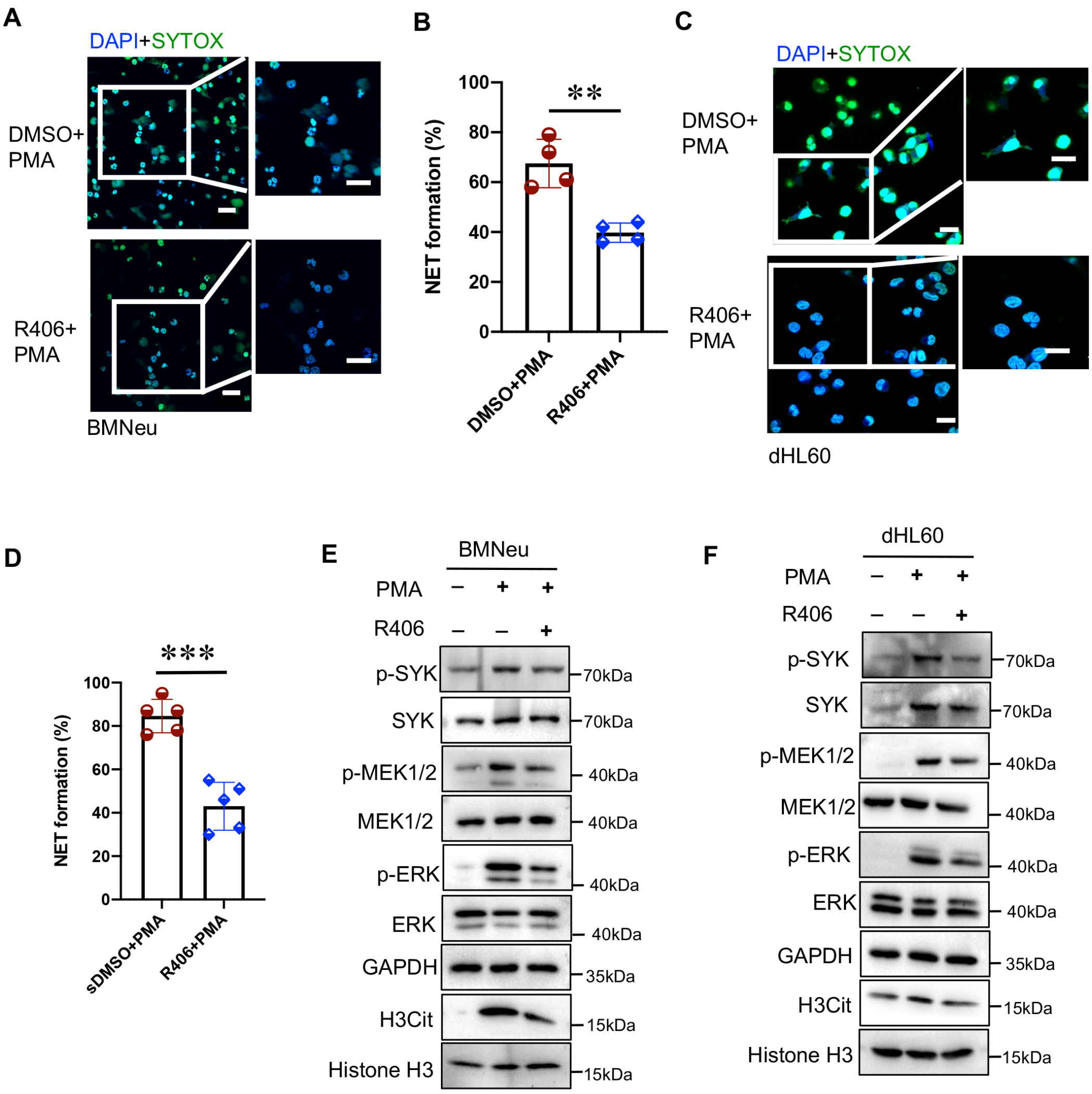

Supplemental Fig. 6. SYK inhibitor R406 reduce Net formation.

(A–D) SYK inhibition with R406 reduces NET formation and H3Cit level in bone marrow neutrophils and dHL60 neutrophils (scale bar, 20um). (E-F) R406 reduces ERK/MEK pathway activation in neutrophils. Data represent mean ± SEM, n ≥ 3 per group. *$p < 0.05$, **$p < 0.01$, ***$p < 0.001$.

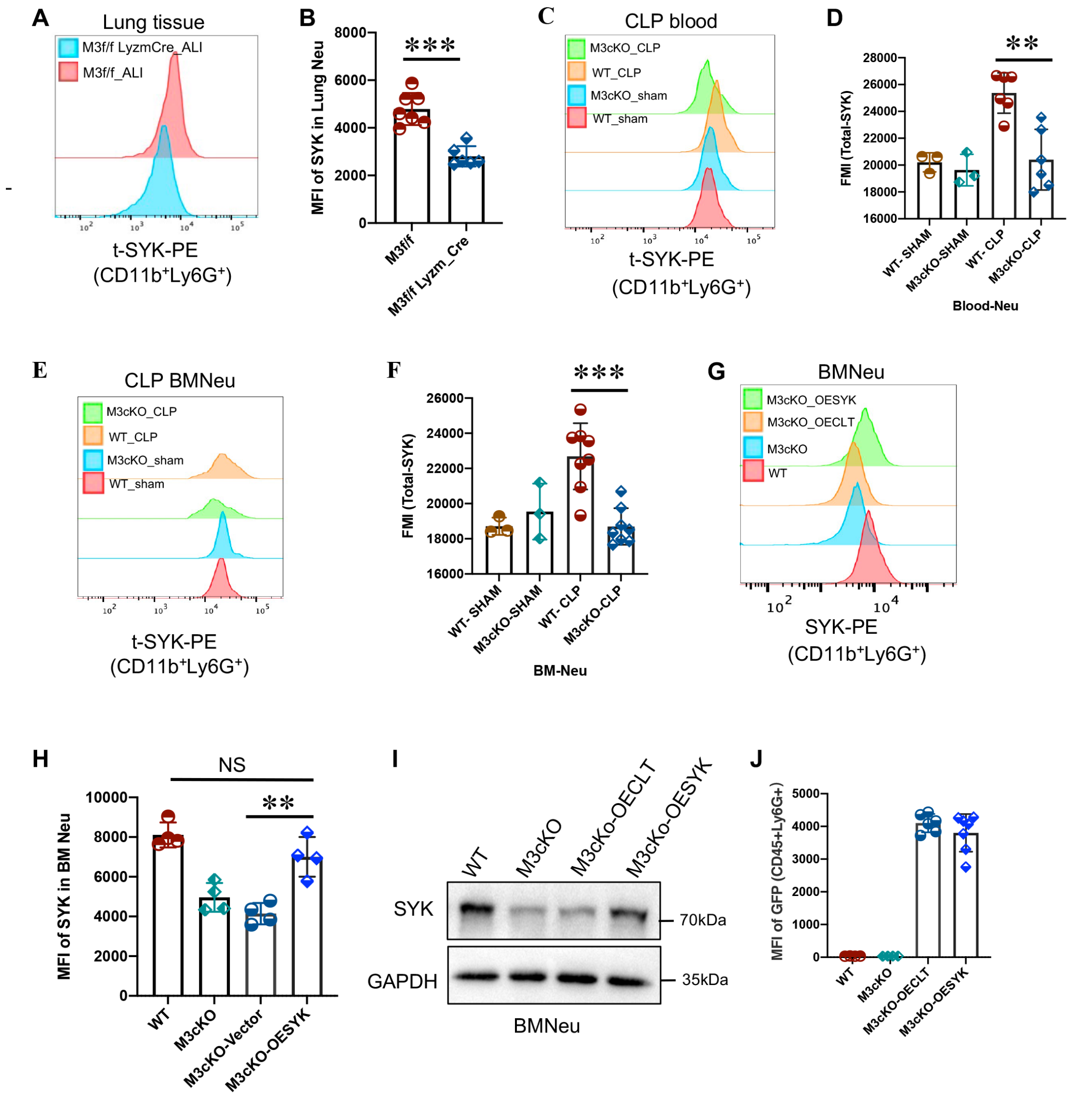

Supplemental Fig. 7. METTL3-dependent SYK expression contributes to sepsis-induced lung injury

(A–B) Flow cytometry of SYK in lung neutrophils of LPS- induced ALI mice. (C–F) Flow cytometry of SYK in lung neutrophils of CLP-sepsis mice. (G-I) SYK expression after SYK overexpression in vivo. (J) GFP expression of SYK lentivirus construct gated on Ly6G+ neutrophil population. Data represent mean ± SEM, $n \geq 3$ per group. $*p < 0.05$, $**p < 0.01$, $***p < 0.001$.

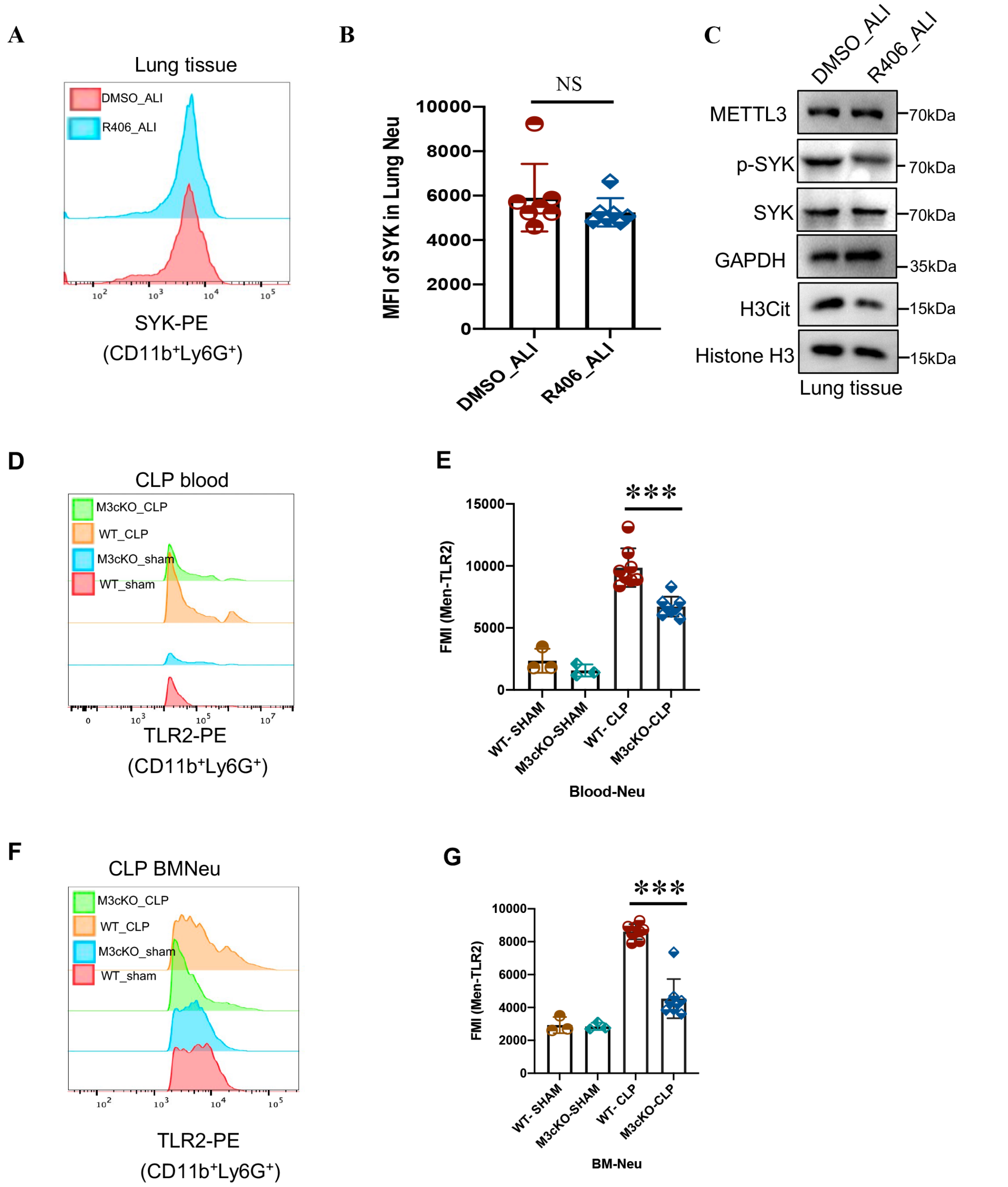

Supplemental Fig. 8. SYK expression promotes sepsis-induced lung injury.

(A-B) SYK expression of lung neutrophil with R406 treatment. (C) R406 inhibits SYK phosphorylation and H3Cit levels in lung tissue. (D–G) Flow cytometry of TLR2 in lung neutrophils of CLP-sepsis mice. Data represent mean ± SEM, n ≥ 3 per group. *p < 0.05, **p < 0.01, ***p < 0.001.

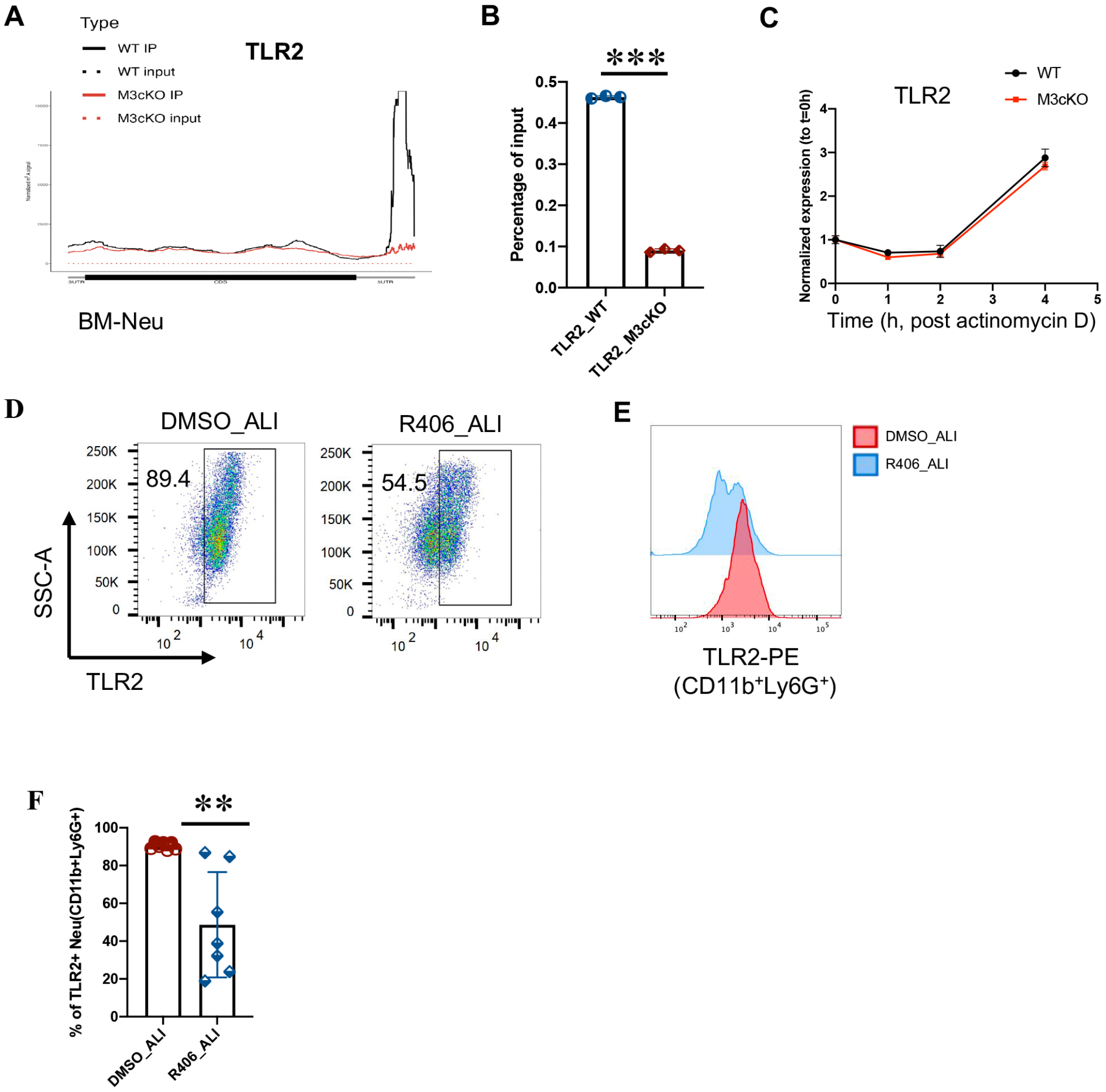

Supplemental Fig. 9. METTL3-dependent SYK expression regulates TLR2 expression in sepsis-induced lung injury.

(A) MeRIP-seq tracks showing m6A peaks in TLR2 Transcripts of WT and Mettl3 cKO neutrophils. (B) MeRIP-qPCR and qPCR of TLR2 transcripts. (C) Level of SYK transcripts in neutrophils treated with the transcription inhibitor actinomycin D. (D-F) Flow cytometry of TLR2 in lung neutrophils after R406 treatment. Data represent mean ± SEM, $n \geq 3$ per group. *$p < 0.05$, **$p < 0.01$, ***$p < 0.001$.

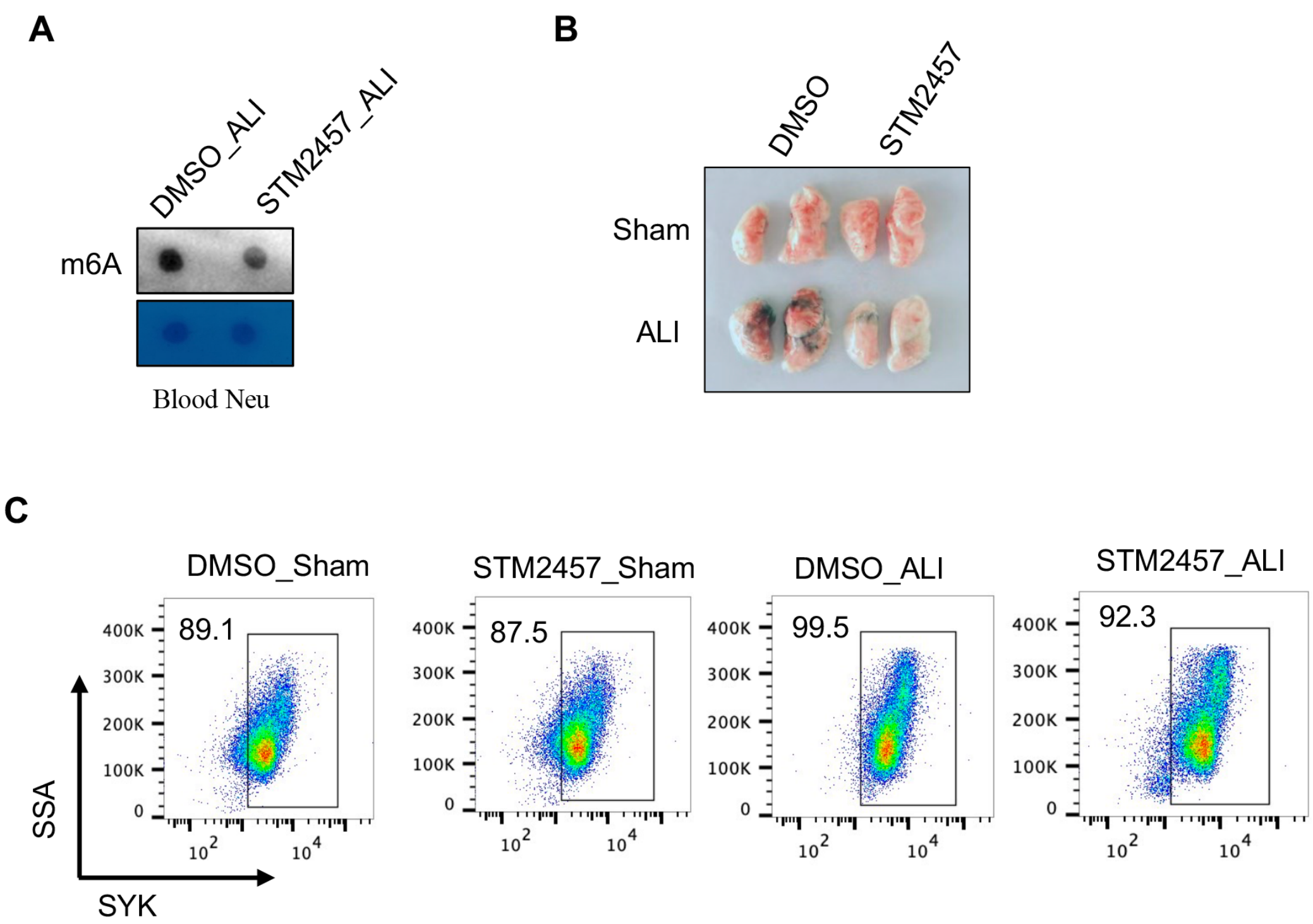

Supplemental Fig. 10. Pharmacological inhibition of METTL3 suppresses NETosis and lung inflammation.

(A) STM2457 reduces m6A level in blood neutrophils. (B) Representative image of Evans blue extravasation in the lungs. (C) Flow cytometry of SYK in lung neutrophils after STM2457 treatment.
