# Supplementary material for: METTL3 promotes neutrophil extracellular trap formation via SYK/ERK/MEK signaling during acute lung injury
Source: J Adv Res. 2026 Jan 22;88:367–79. doi: 10.1016/j.jare.2026.01.044 (PMC13669593; doi:10.1016/j.jare.2026.01.044)

Fig. 1

J

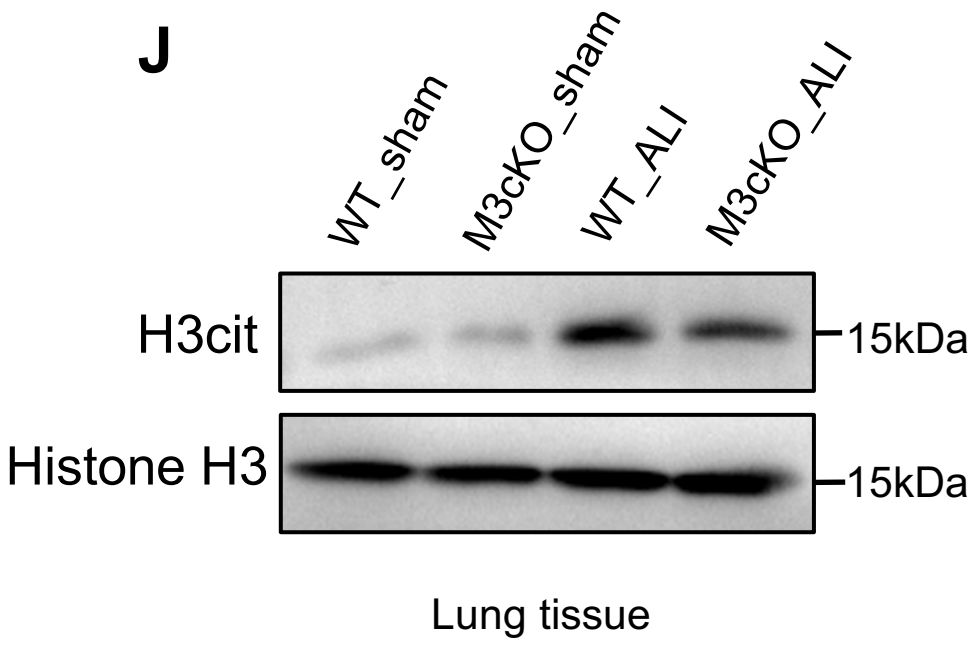

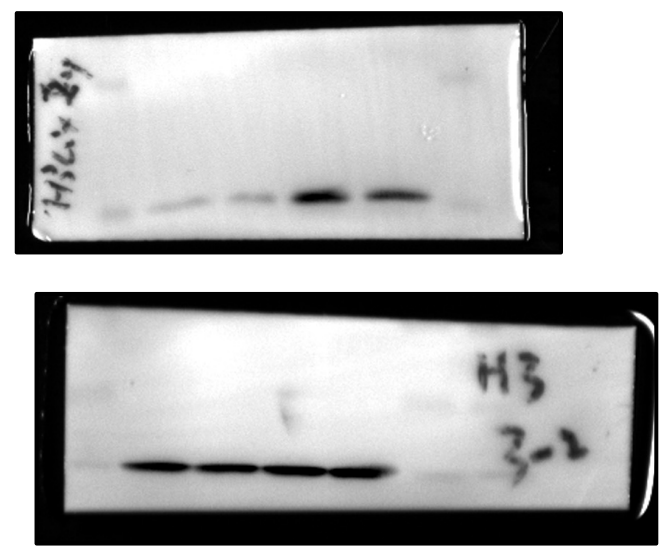

Sup Fig. 1

B

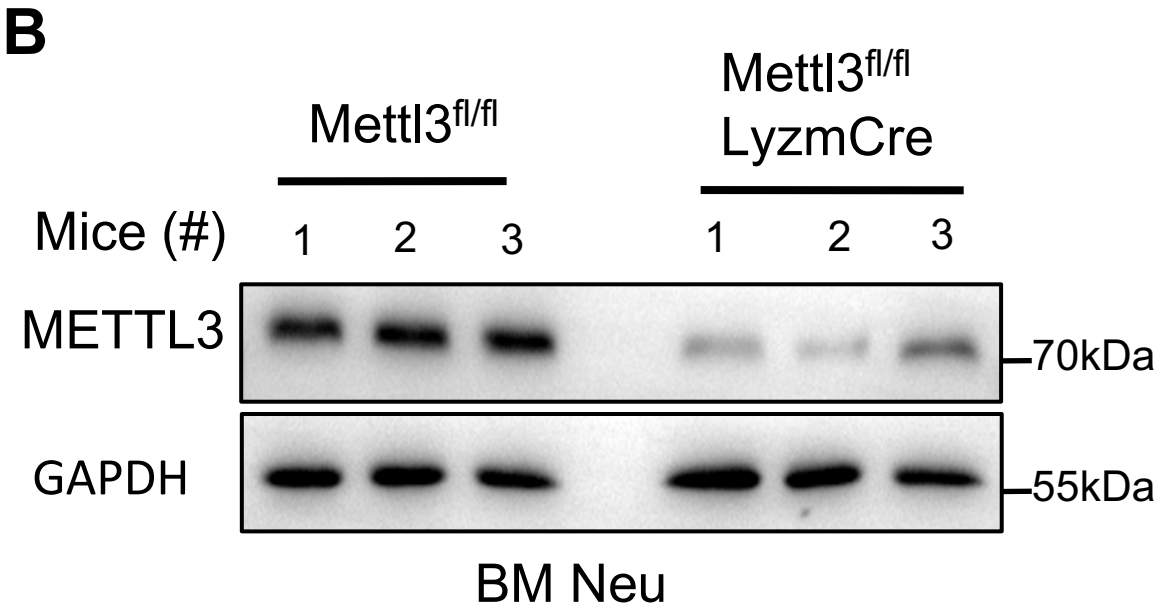

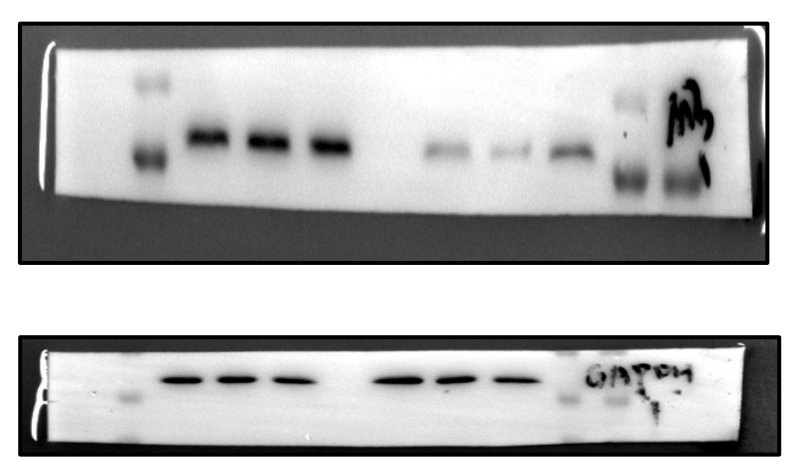

H

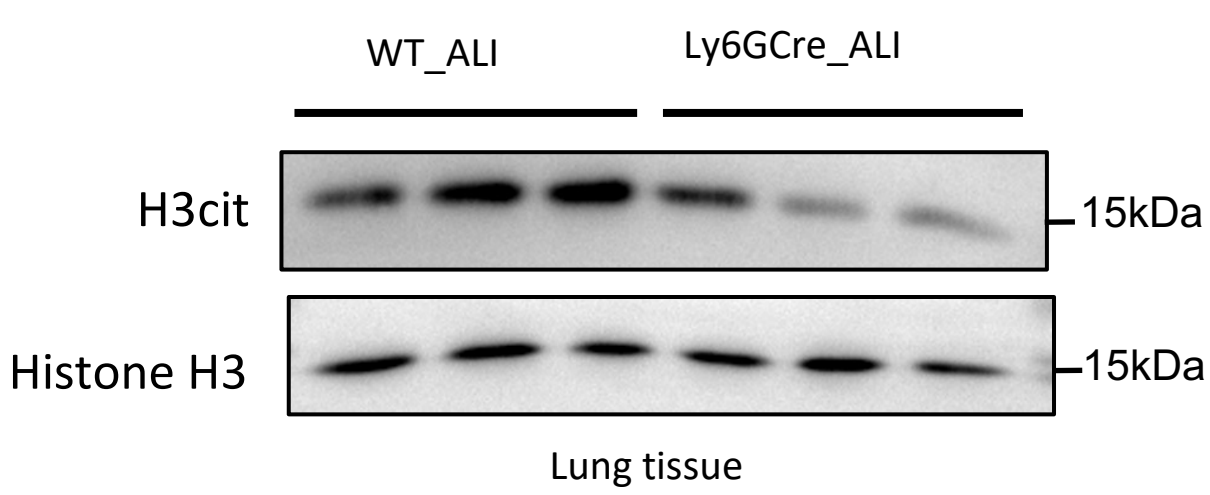

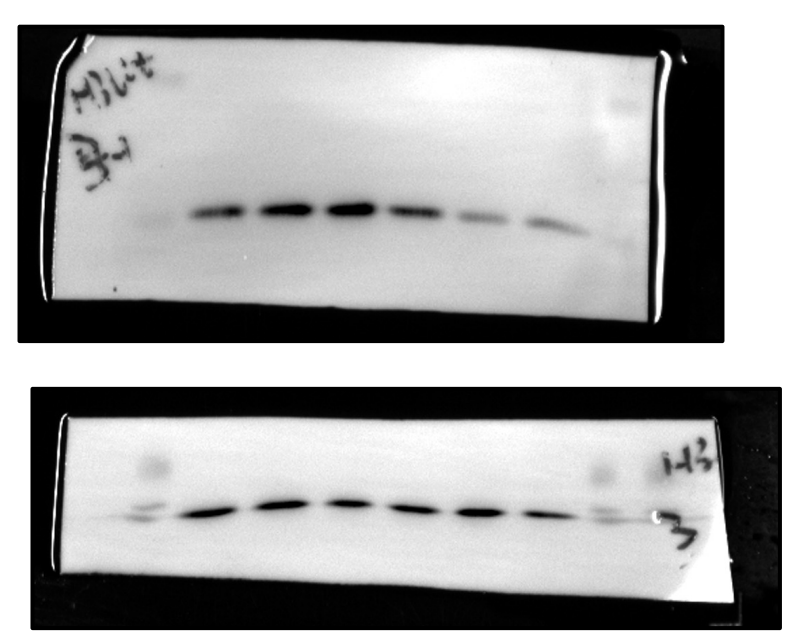

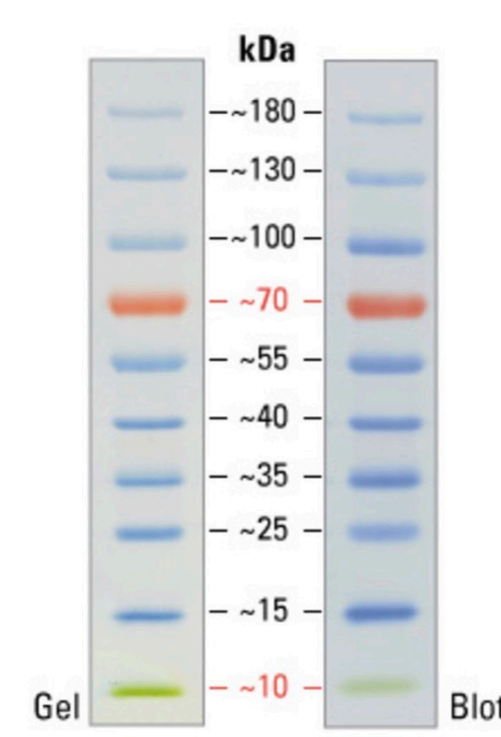

Fig. 2

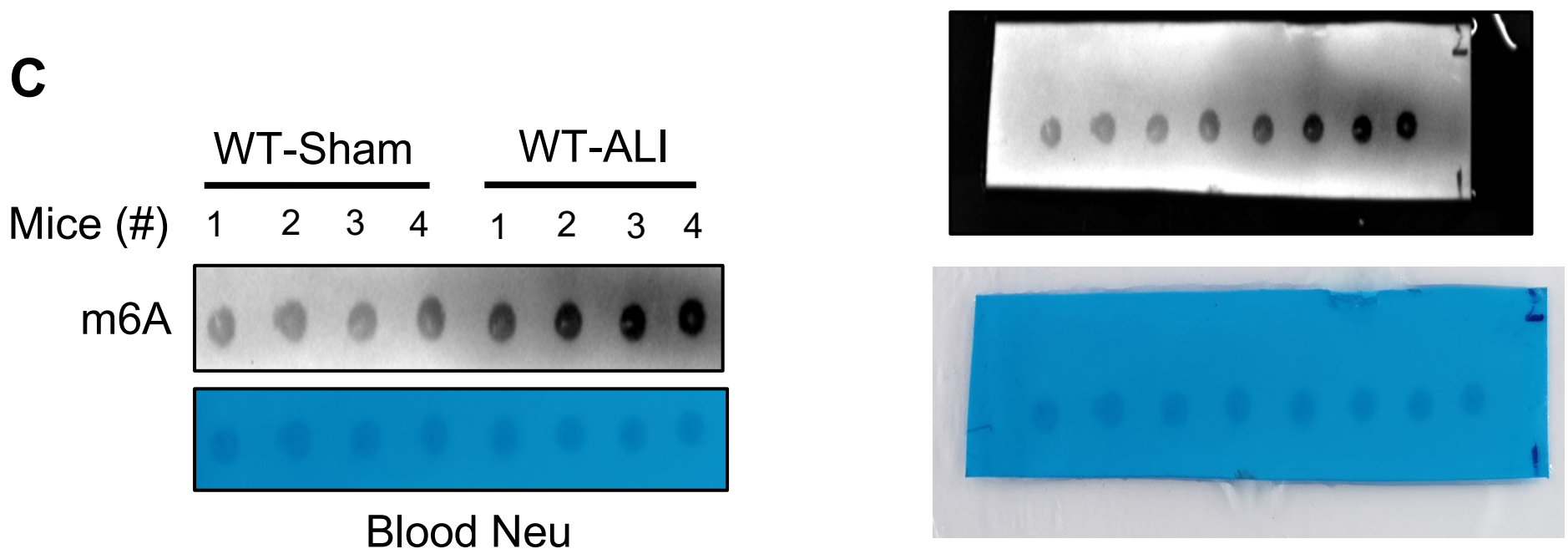
C
WT-Sham
WT-ALI
Mice (#)
1 2 3 4 1 2 3 4
m6A
Blood Neu

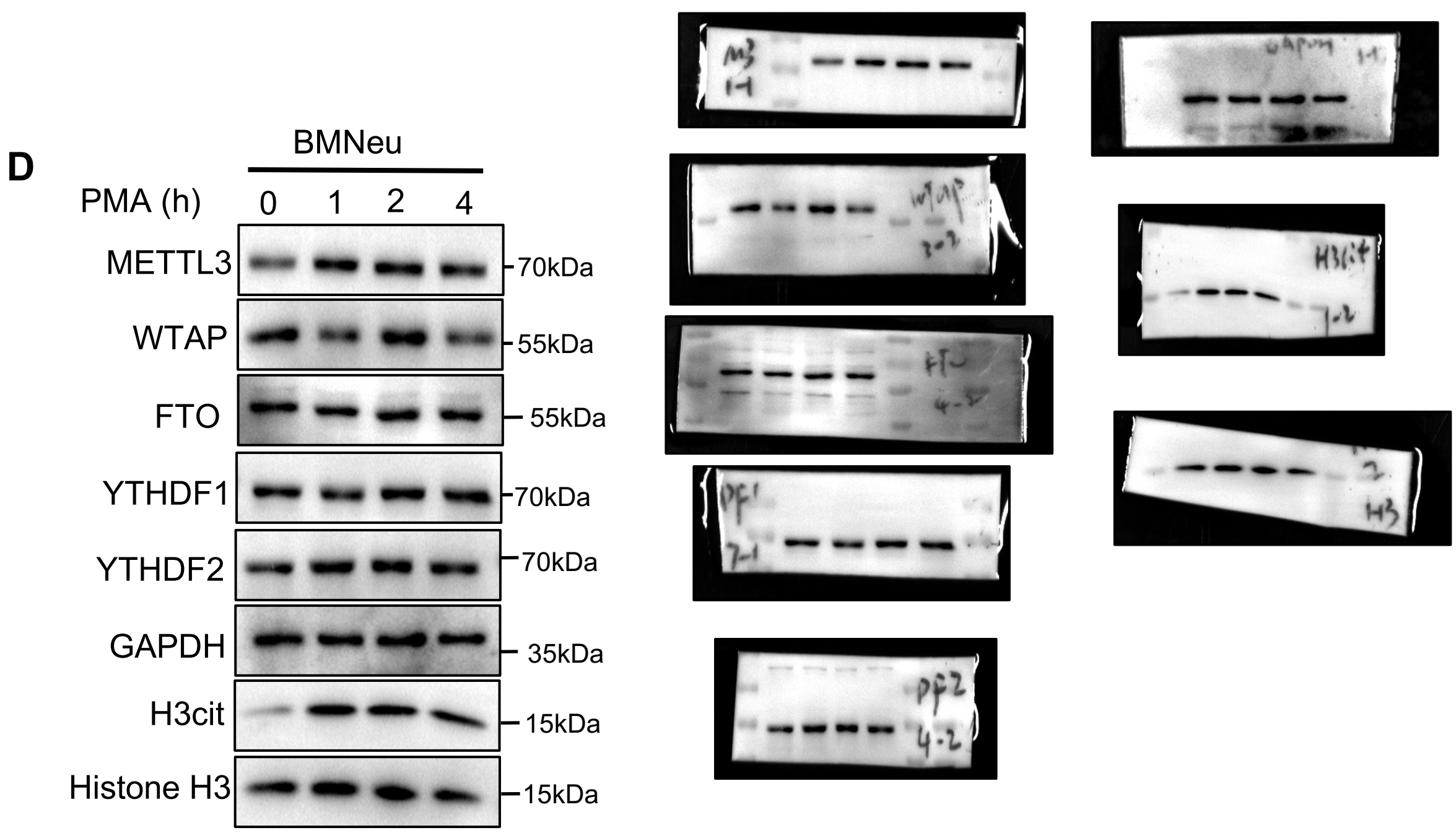
D
BMNeu
PMA (h)
0 1 2 4
METTL3
70kDa
WTAP
55kDa
FTO
55kDa
YTHDF1
70kDa
YTHDF2
70kDa
GAPDH
35kDa
H3cit
15kDa
Histone H3
15kDa

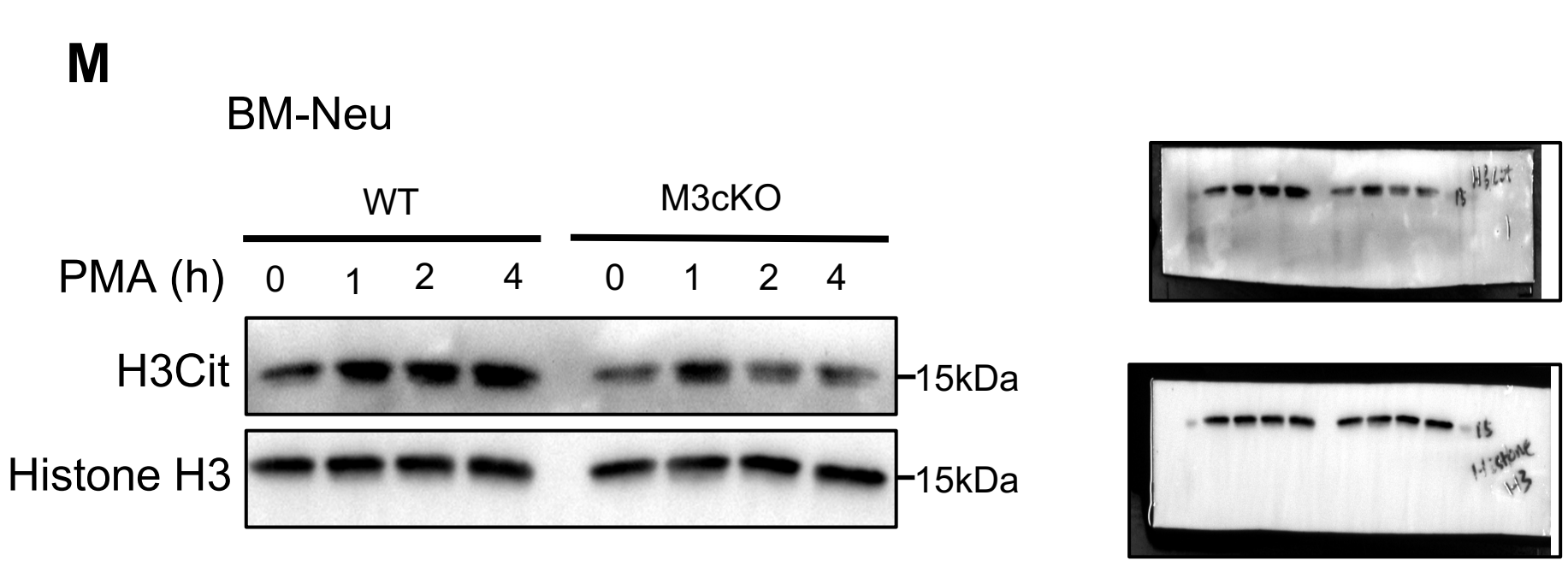
M
BM-Neu
WT
M3cKO
PMA (h)
0 1 2 4 0 1 2 4
H3Cit
15kDa
Histone H3
15kDa

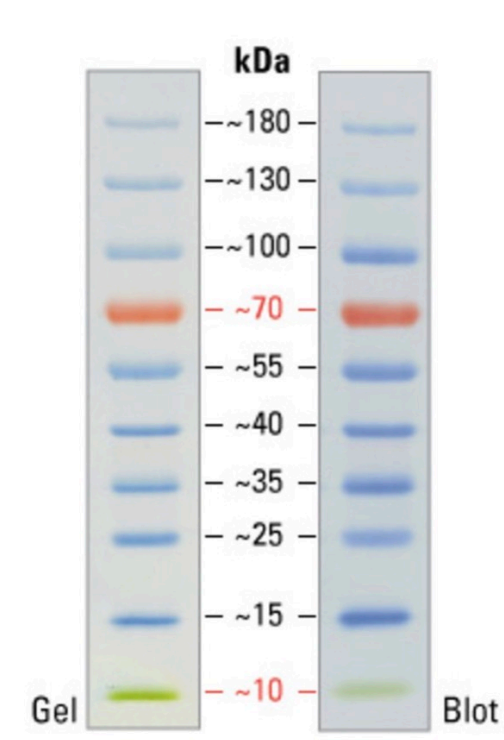
kDa
~180
~130
~100
~70
~55
~40
~35
~25
~15
~10
Gel
Blot

Sup Fig. 3

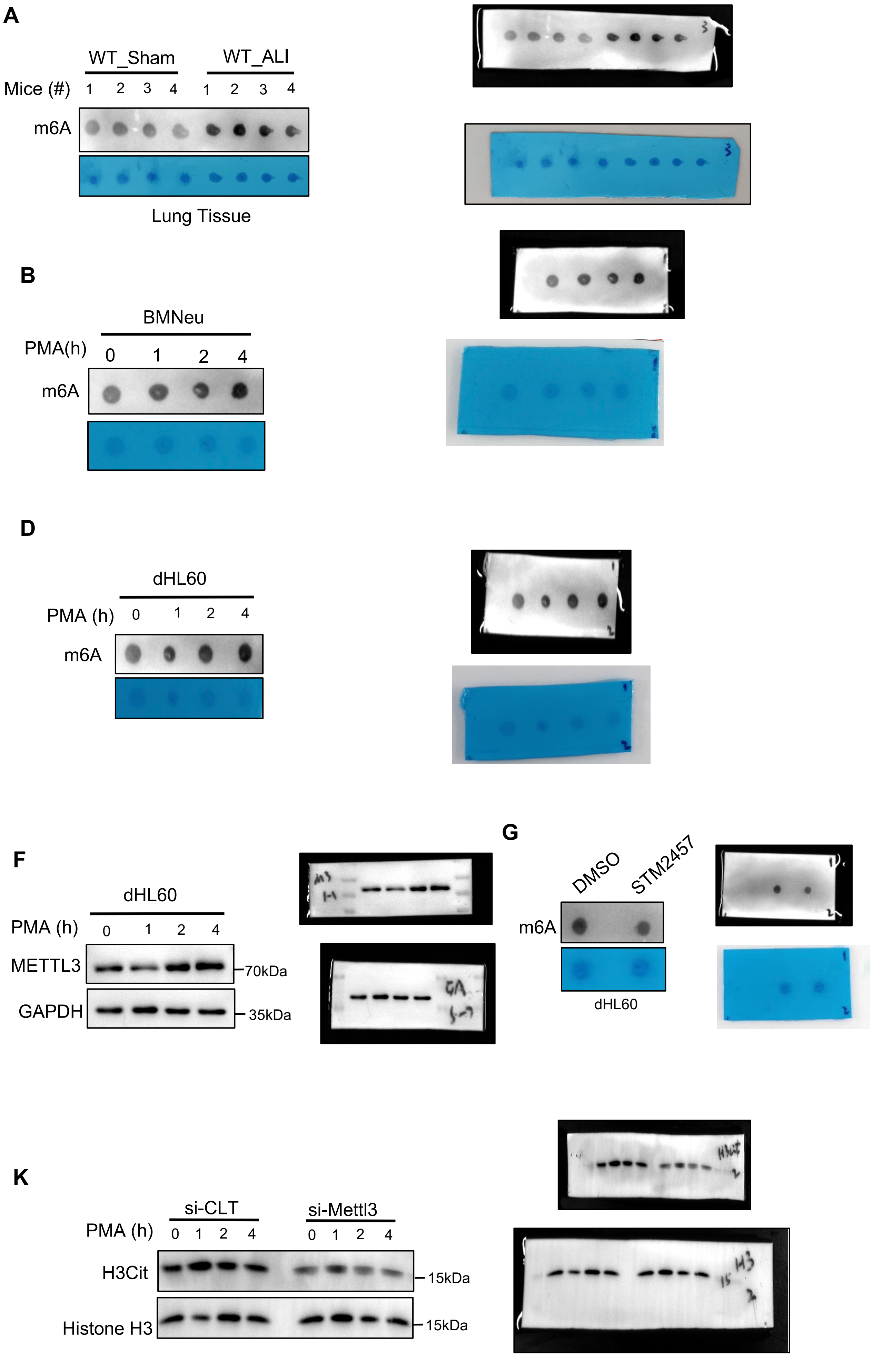

A
WT_Sham
WT_ALI
Mice (#)
1 2 3 4 1 2 3 4
m6A
Lung Tissue
B
BMNeu
PMA(h)
0 1 2 4
m6A
D
dHL60
PMA (h)
0 1 2 4
m6A
F
dHL60
PMA (h)
0 1 2 4
METTL3
70kDa
GAPDH
35kDa
G
DMSO
STM2457
m6A
dHL60
K
si-CLT
si-Mettl3
PMA (h)
0 1 2 4 0 1 2 4
H3Cit
15kDa
Histone H3
15kDa

Fig. 3

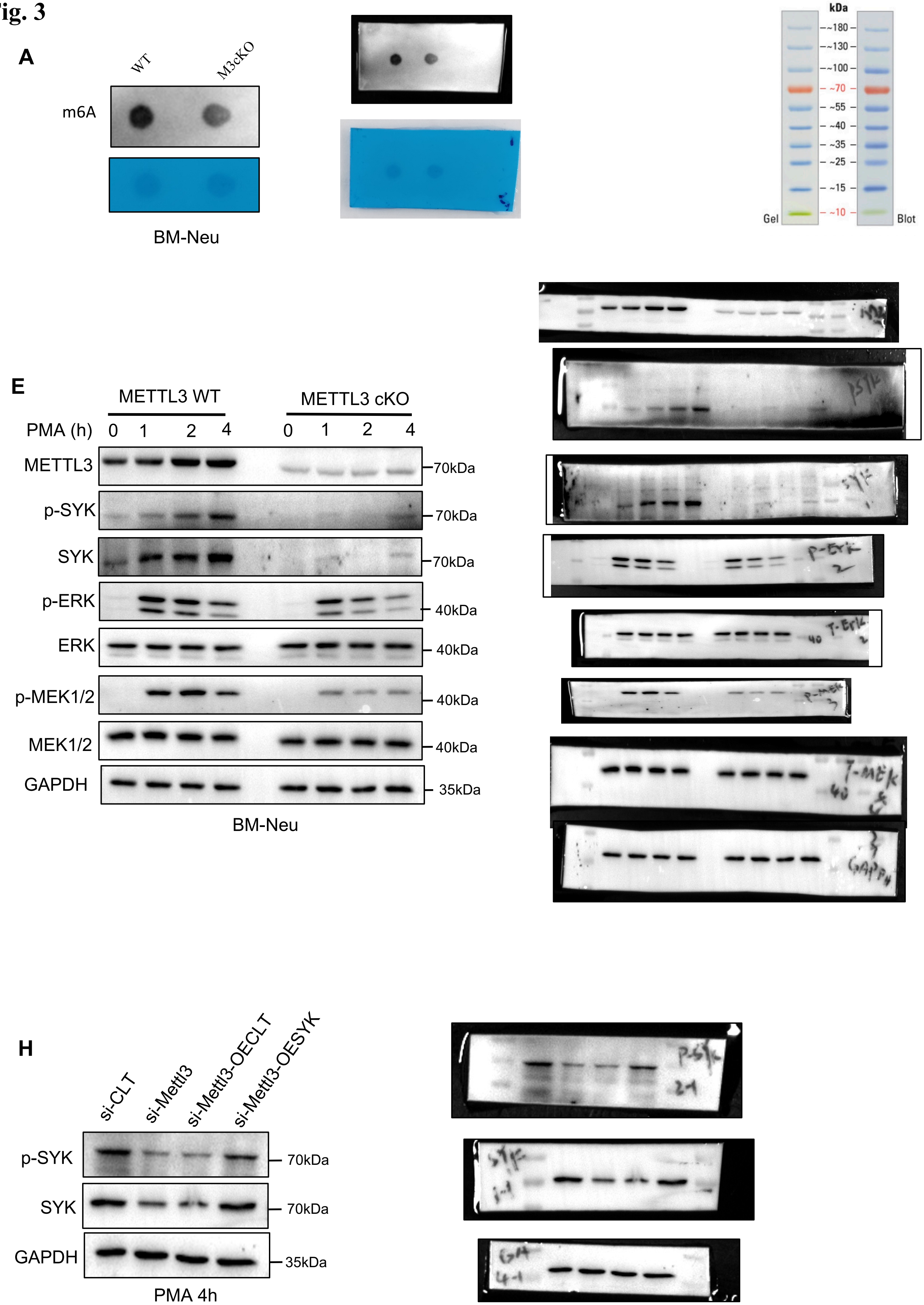
A
WT
M3cKO
m6A
BM-Neu
kDa
~180
~130
~100
~70
~55
~40
~35
~25
~15
~10
Gel
Blot
E
METTL3 WT
METTL3 cKO
PMA (h)
0
1
2
4
0
1
2
4
METTL3
p-SYK
SYK
p-ERK
ERK
p-MEK1/2
MEK1/2
GAPDH
70kDa
70kDa
70kDa
40kDa
40kDa
40kDa
40kDa
35kDa
BM-Neu
H
si-CLT
si-Mettl3
si-Mettl3-OECLT
si-Mettl3-OESYK
p-SYK
SYK
GAPDH
70kDa
70kDa
35kDa
PMA 4h

Sup Fig. 5

B

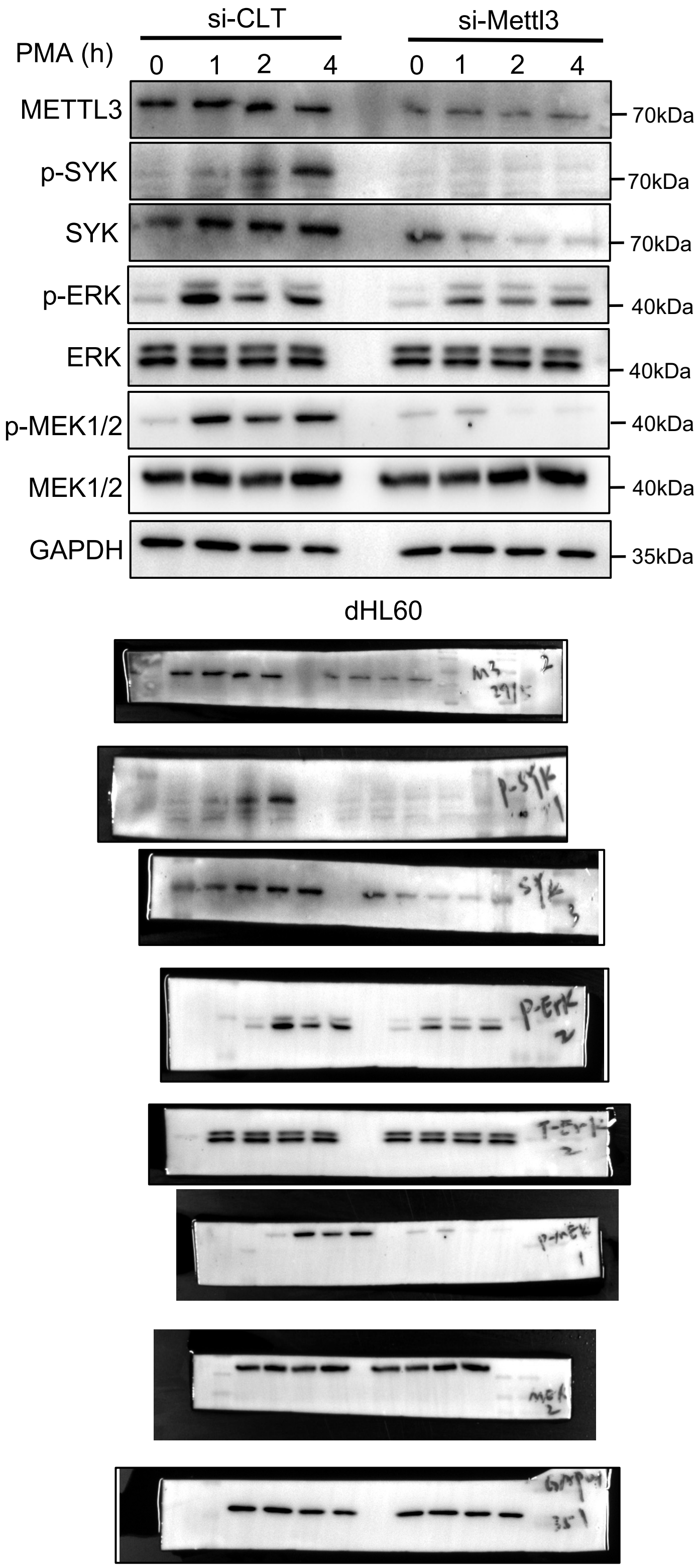

si-CLT
si-Mettl3
PMA (h)
0 1 2 4 0 1 2 4
METTL3
p-SYK
SYK
p-ERK
ERK
p-MEK1/2
MEK1/2
GAPDH
70kDa
70kDa
70kDa
40kDa
40kDa
40kDa
40kDa
35kDa
dHL60

C

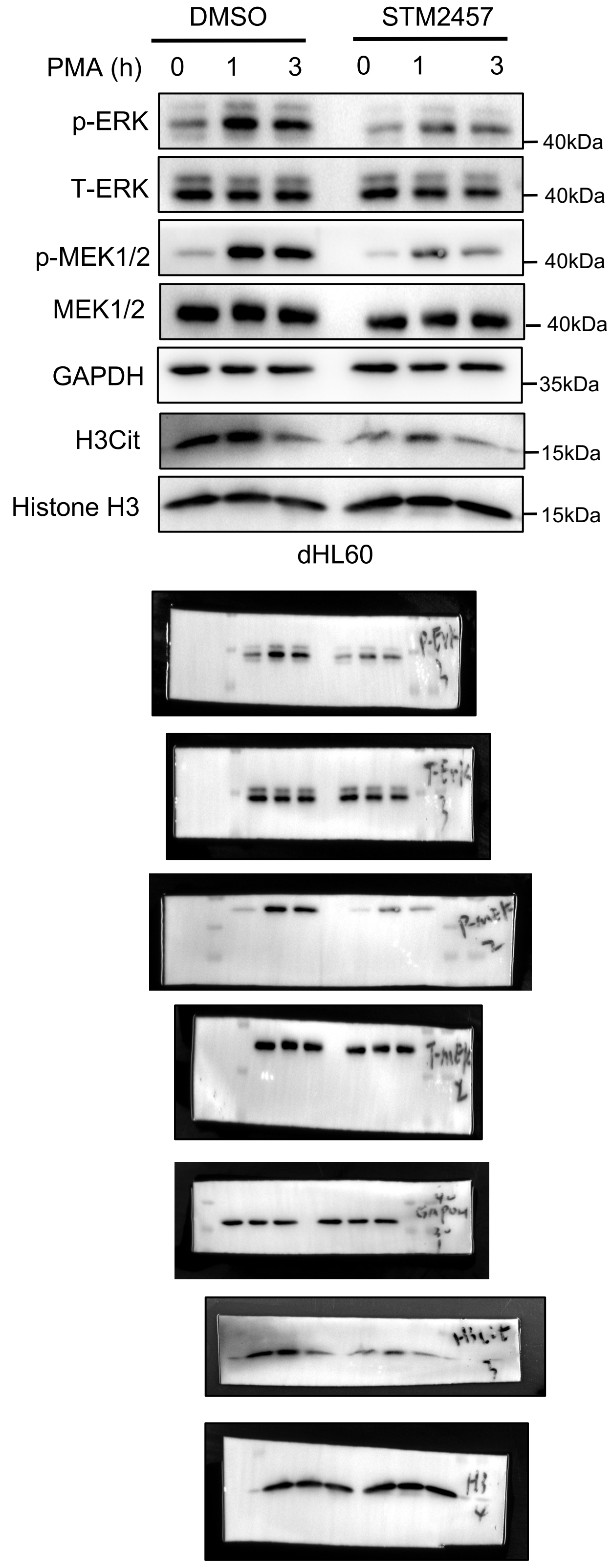

DMSO
STM2457
PMA (h)
0 1 3 0 1 3
p-ERK
T-ERK
p-MEK1/2
MEK1/2
GAPDH
H3Cit
Histone H3
40kDa
40kDa
40kDa
40kDa
35kDa
15kDa
15kDa
dHL60

D

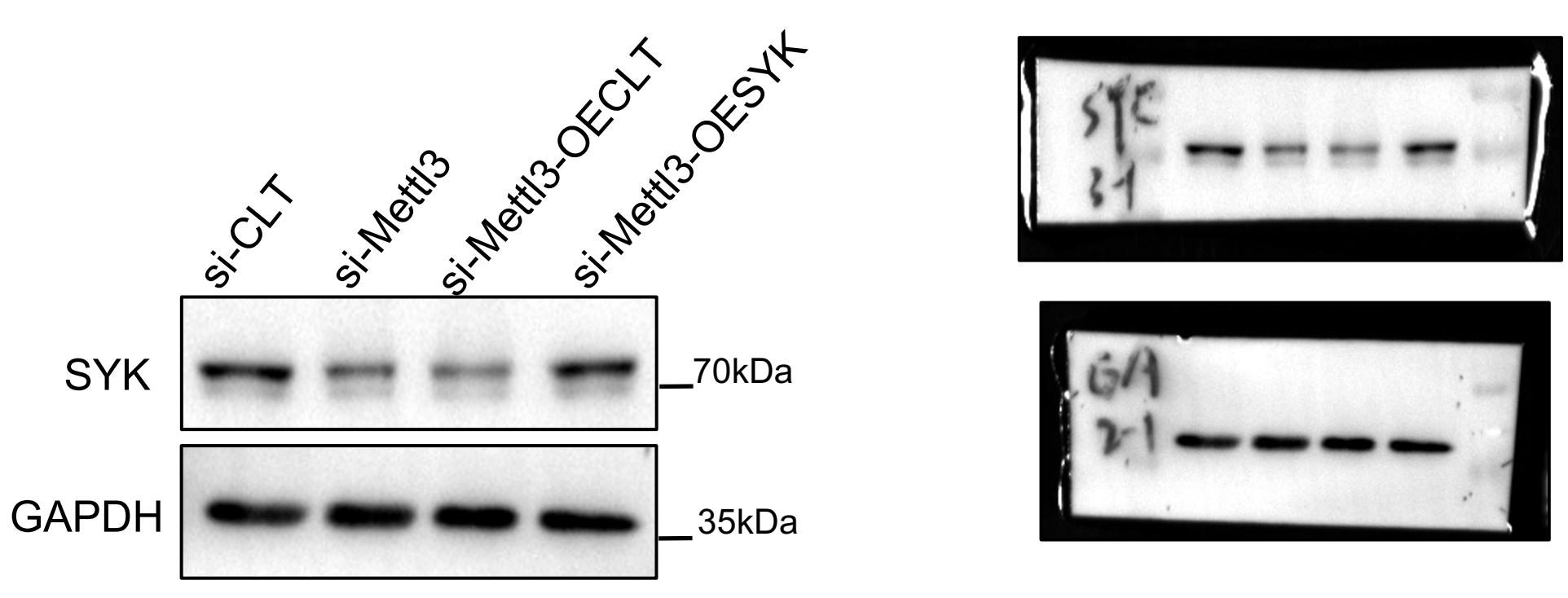

si-CLT
si-Mettl3
si-Mettl3-OECLT
si-Mettl3-OESYK
SYK
GAPDH
70kDa
35kDa

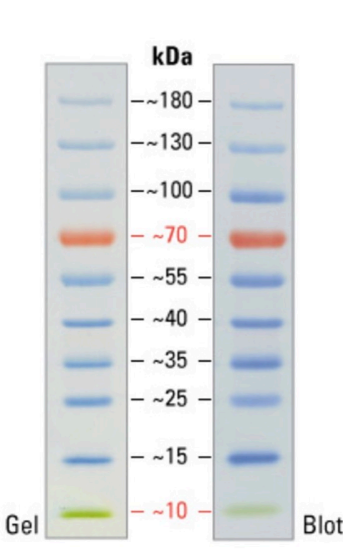

kDa
~180
~130
~100
~70
~55
~40
~35
~25
~15
~10
Gel
Blot

Sup Fig. 6

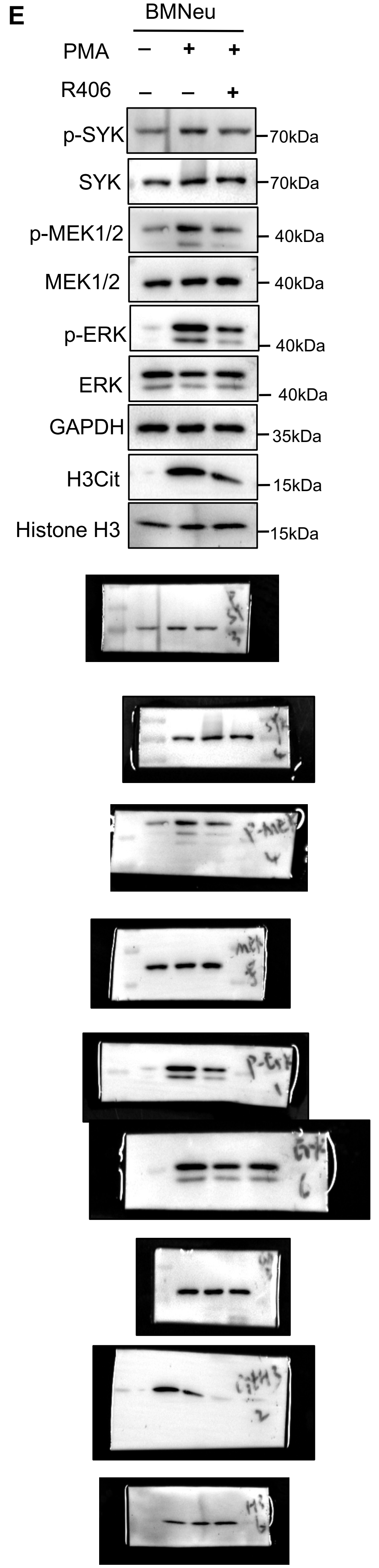
E
BMNeu
PMA − + +
R406 − − +
p-SYK
SYK
p-MEK1/2
MEK1/2
p-ERK
ERK
GAPDH
H3Cit
Histone H3
70kDa
70kDa
40kDa
40kDa
40kDa
40kDa
35kDa
15kDa
15kDa

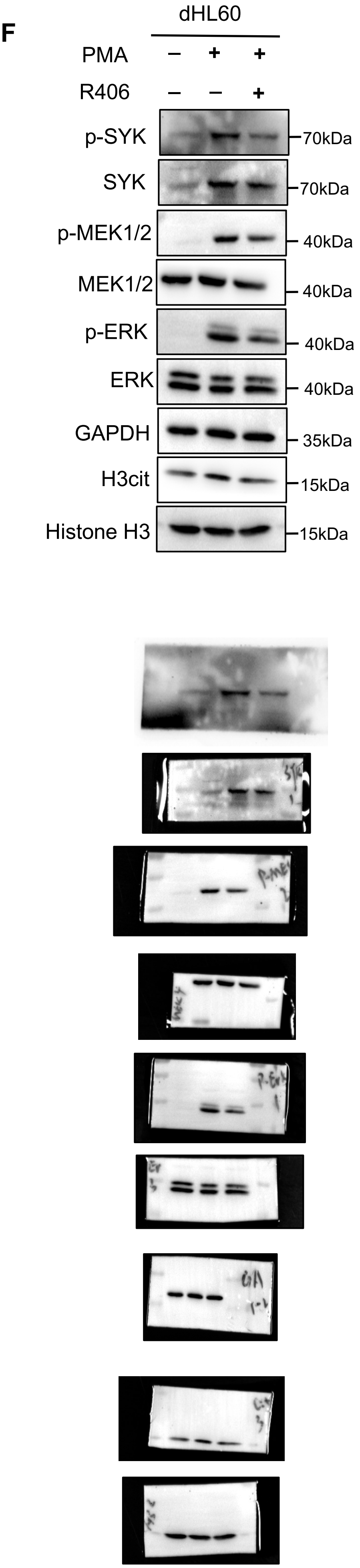
F
dHL60
PMA − + +
R406 − − +
p-SYK
SYK
p-MEK1/2
MEK1/2
p-ERK
ERK
GAPDH
H3cit
Histone H3
70kDa
70kDa
40kDa
40kDa
40kDa
40kDa
35kDa
15kDa
15kDa

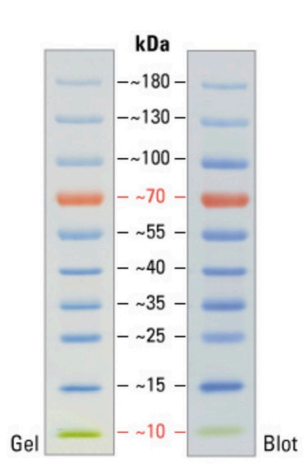
kDa
~180
~130
~100
~70
~55
~40
~35
~25
~15
~10
Gel
Blot

Fig. 4

G

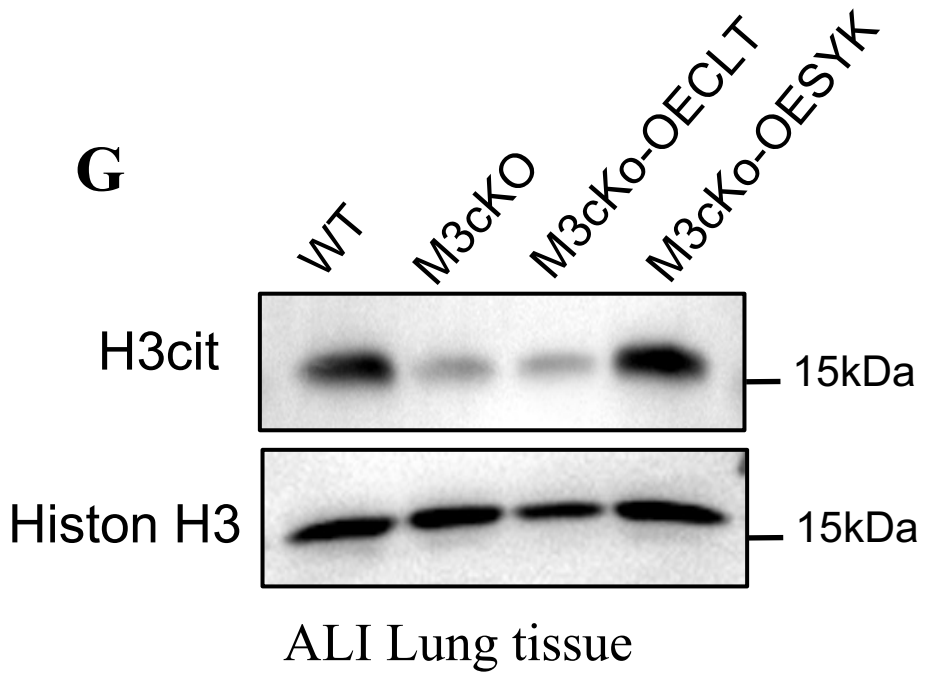

WT
M3cKO
M3cKo-OECLT
M3cKo-OESYK
H3cit
15kDa
Histon H3
15kDa
ALI Lung tissue

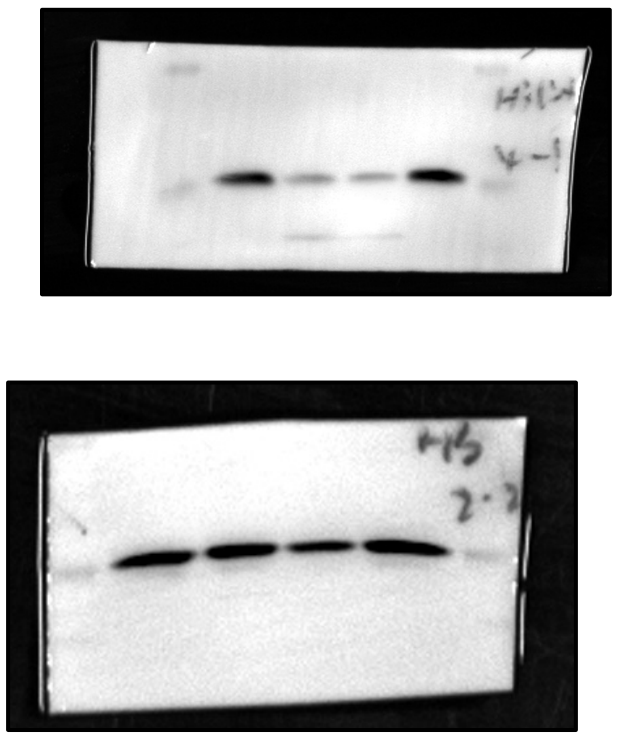

N

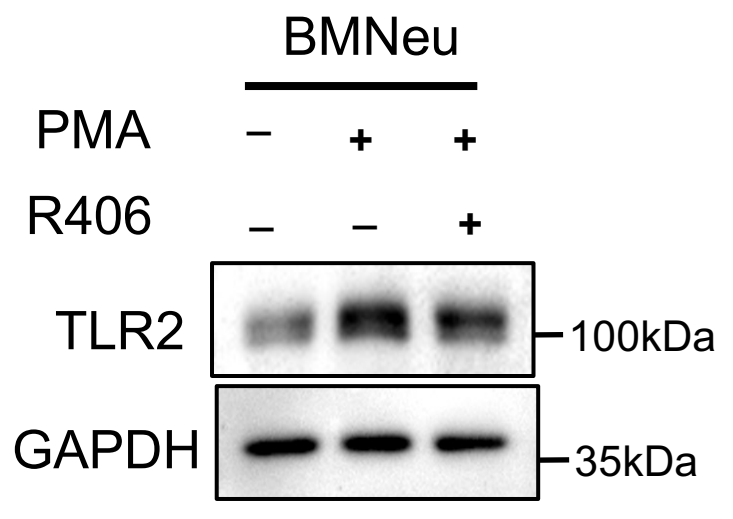

BMNeu
PMA
–
+
+
R406
–
–
+
TLR2
100kDa
GAPDH
35kDa

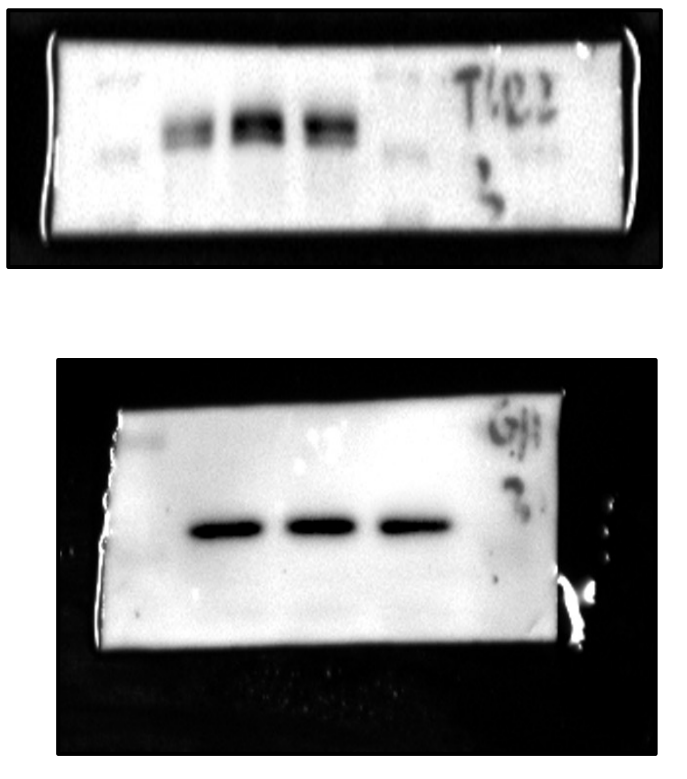

P

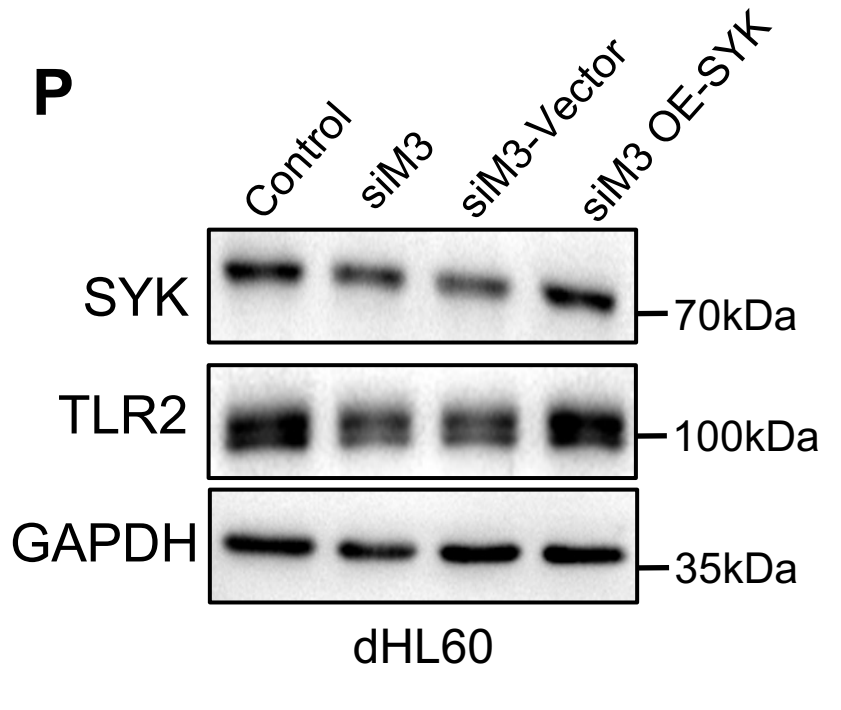

Control
siM3
siM3-Vector
siM3 OE-SYK
SYK
70kDa
TLR2
100kDa
GAPDH
35kDa
dHL60

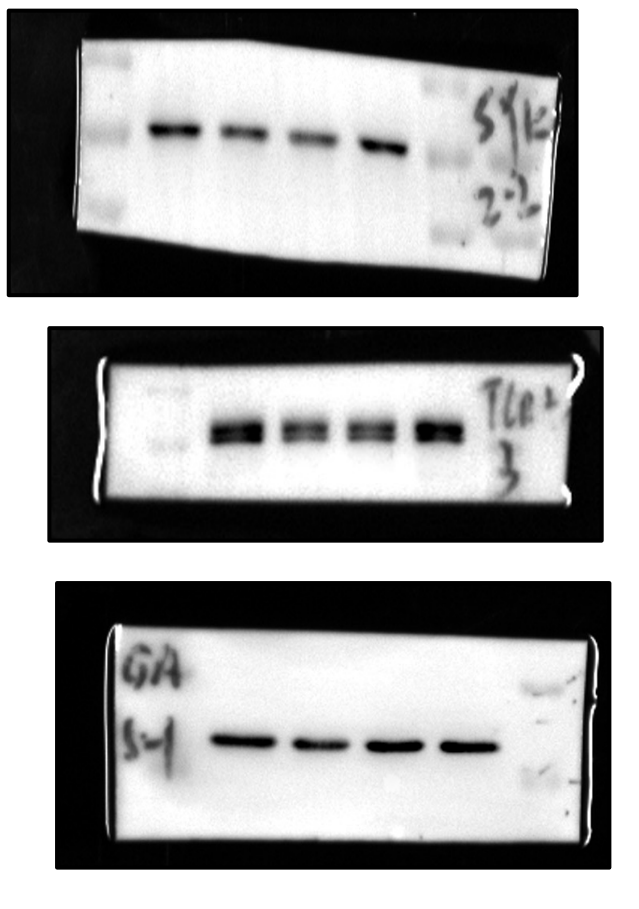

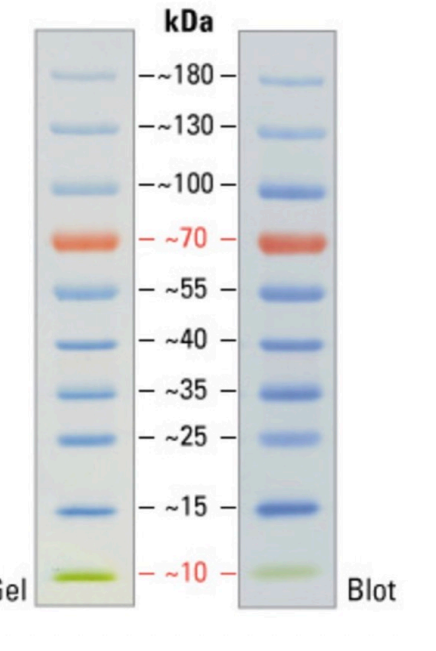

kDa
~180
~130
~100
~70
~55
~40
~35
~25
~15
~10
Gel
Blot

Sup Fig. 7

I

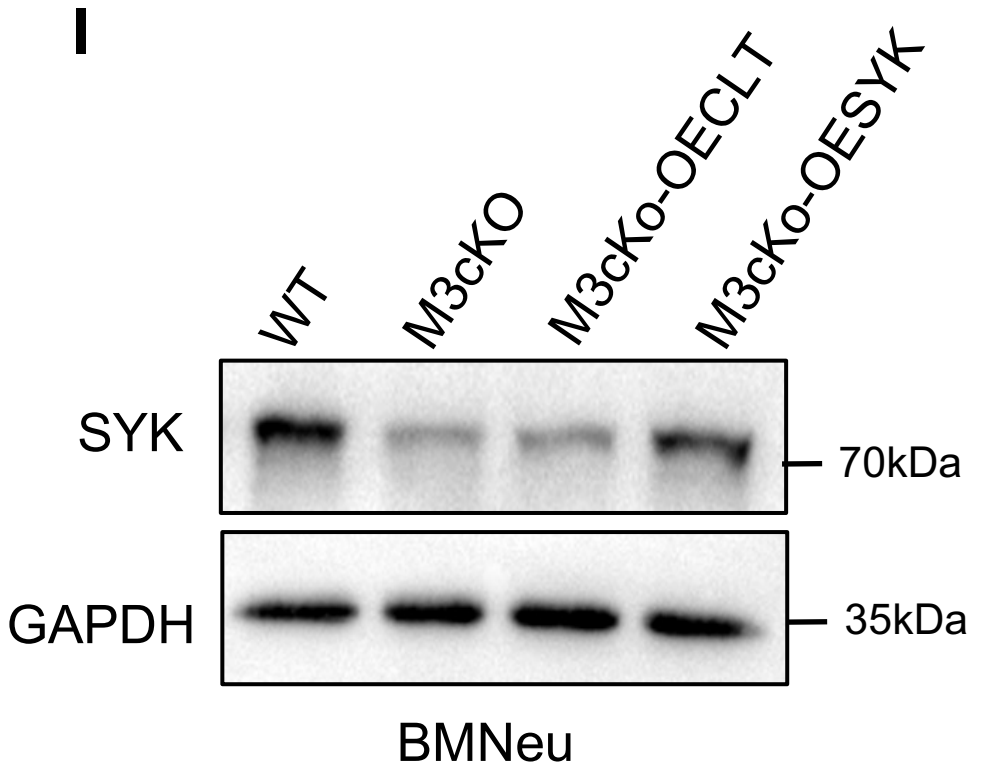

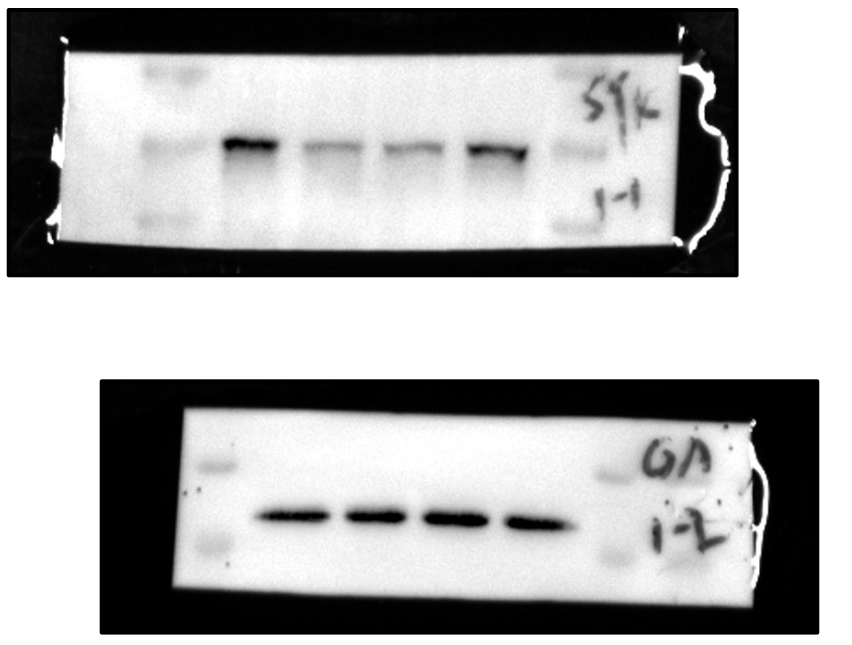

Sup Fig. 8

E

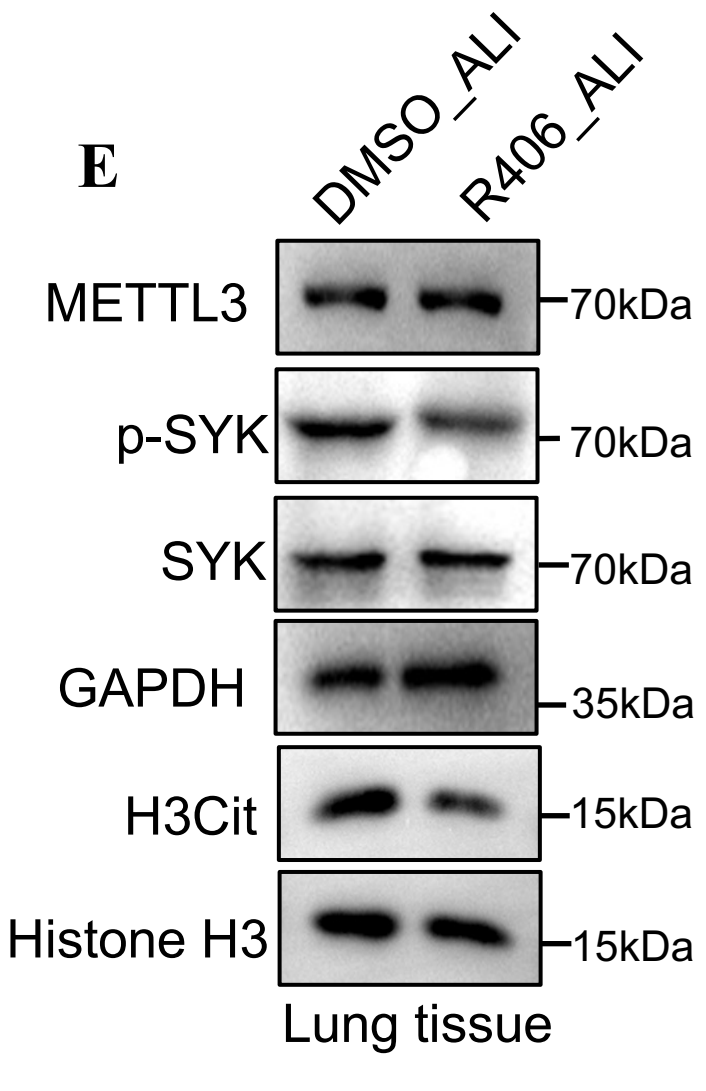

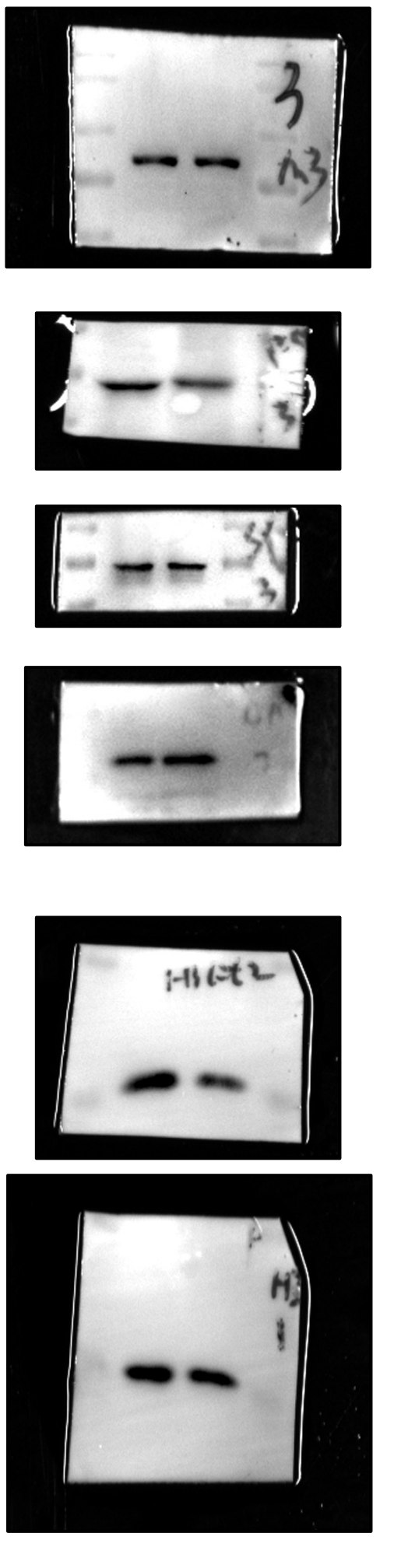

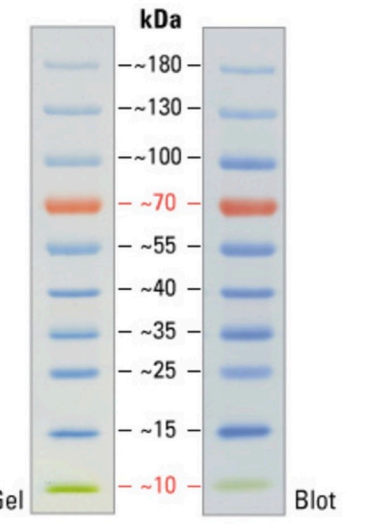

Fig. 6

A

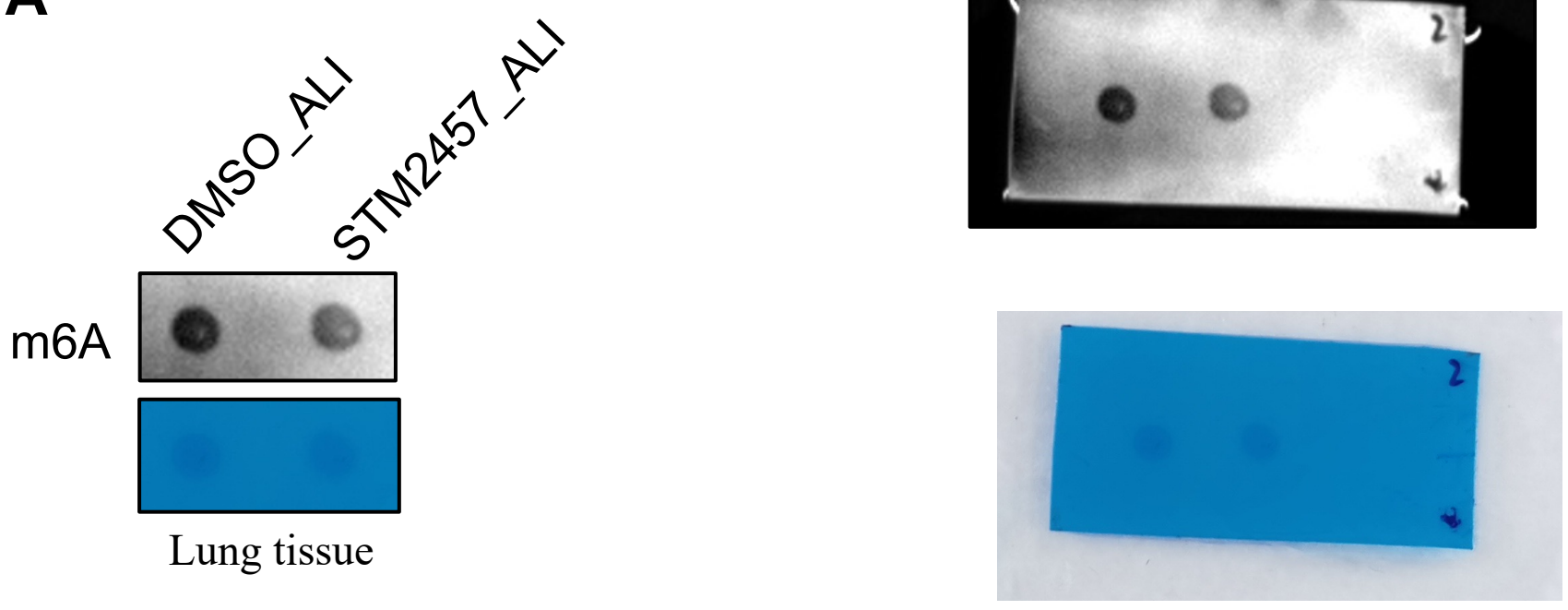

K

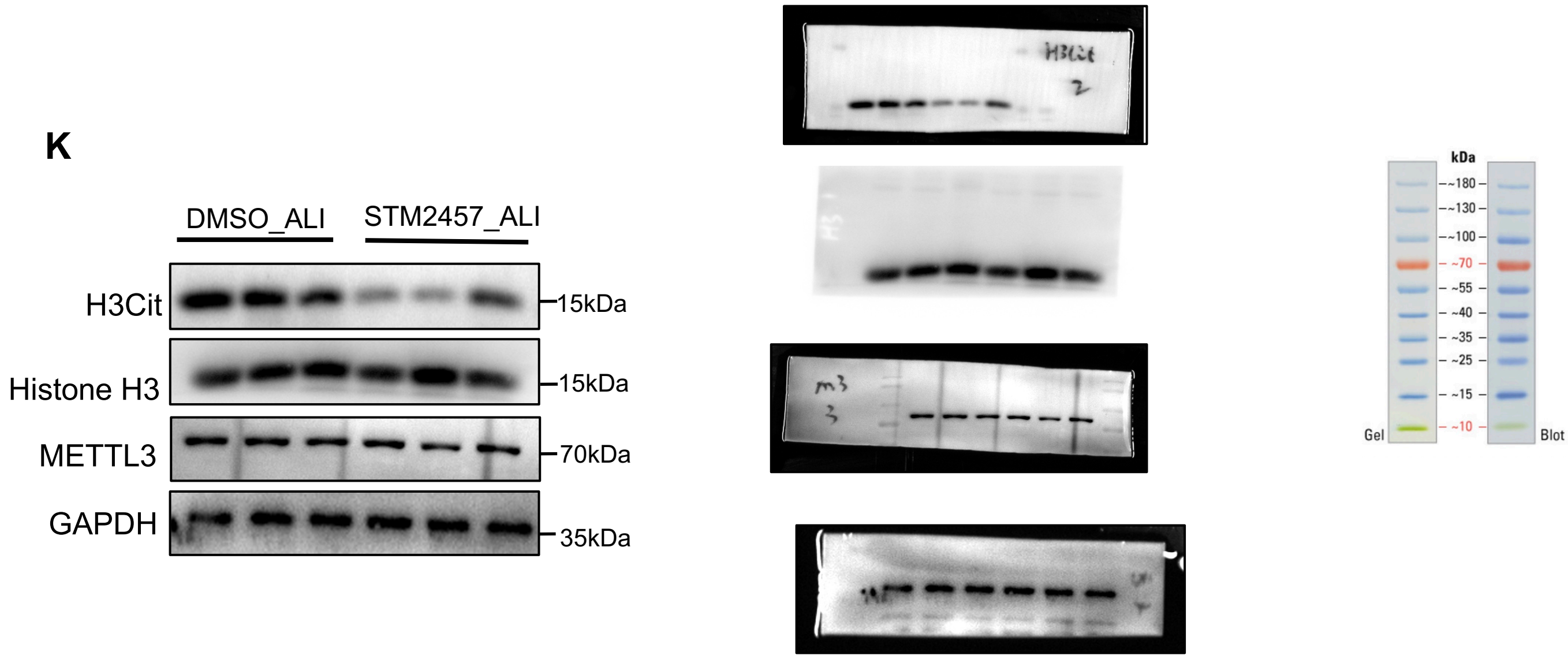

Sup Fig. 10

A

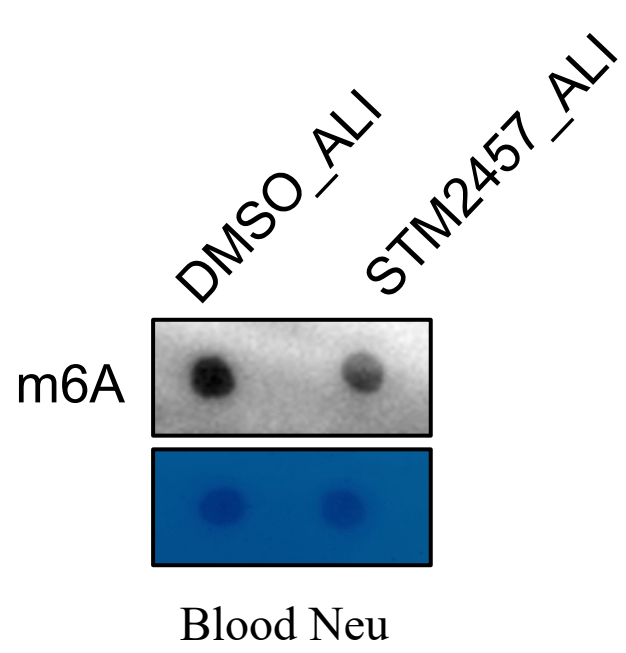

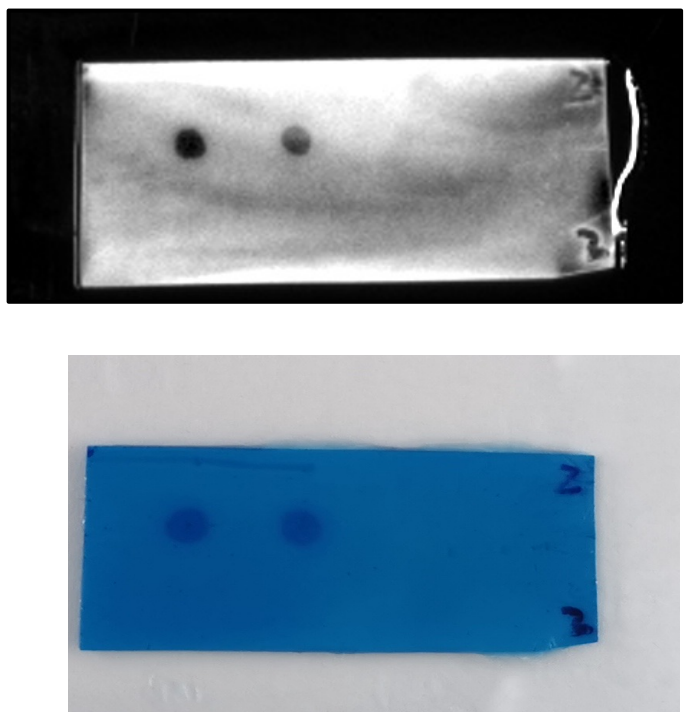

Supplement: Supplementary file 2 [file mmc4.pdf]
