# Supplementary material for: METTL3 promotes neutrophil extracellular trap formation via SYK/ERK/MEK signaling during acute lung injury
Source: J Adv Res. 2026 Jan 22;88:367–79. doi: 10.1016/j.jare.2026.01.044 (PMC13669593; doi:10.1016/j.jare.2026.01.044)
Supplement: Supplementary file 3 [file mmc3.docx]

**Key resources table**

| REAGENT or RESOURCE | SOURCE | IDENTIFIER |
| --- | --- | --- |
| Antibodies | | |
| APC Rat Anti-Mouse Ly-6G | BD | 560599 |
| CD11b FITC M1/70 | BD Pharmingen | 557396 |
| CD45 Monoclonal Antibody (30-F11), PE, eBioscience™ | Invitrogen | 12-0451-82 |
| PerCP/Cyanine5.5 anti-mouse CD45 Antibody | Biolegend | 103132 |
| PE anti-Syk Antibody | Biolegend | 646003 |
| PE anti-mouse CD282 (TLR2) Antibody | Biolegend | 148603 |
|  |  |  |
| Anti-N6-methyladenosine (m6A) antibody (ab232905) | abcam | ab232905) |
| Antibody-METTL3 antibody（EPR18810） | abcam | AB195352 |
| β-Actin (8H10D10) Mouse mAb #3700 | CST | #3700 |
| GAPDH Monoclonal antibody | ProteinTech | 60004-1-Ig |
| Anti-Syk (phospho Y352) +ZAP70 (phospho Y319) antibody [EPR26232-39] (ab300398) | abcam | ab300398 |
| Syk (D3Z1E) XP® Rabbit mAb #13198 | CST | 13198T |
| Anti-MEK1 + MEK2 antibody [EPR16667] (ab178876) | abcam | ab178876 |
| Anti-MEK1 (phospho S221) + MEK2 (phospho S221) antibody [MEK12S221-D3] (ab278564) | abcam | ab278564 |
| TLR2 Polyclonal antibody | ProteinTech | 17236-1-AP |
| Anti-Myeloperoxidaseantibody [EPR20257] (ab208670) | abcam | ab208670 |
| TLR4 antibody (76B357.1): sc-52962 | Santa Cruz Biotechnology | 52962 |
| FTO Polyclonal antibody | proteintech | 27226-1-AP |
| YTHDF1 Polyclonal antibody | proteintech | 17479-1-AP |
| YTHDF2 Polyclonal antibody | proteintech | 24744-1-AP |
| WTAP Monoclonal antibody | proteintech | 60188-1-Ig |
| YTHDC2 Recombinant antibody | proteintech | 83970-1-RR |
|  |  |  |
| Bacterial and virus strains | | |
| GV293-hMETTL3-RNAi (hU6-MCS-Cbh-gcGFP-IRES-Puro) | Shanghai GeneChem co.,Ltd | GIEL0331192 |
| LV5-EF1α-hSYK-GFP&Puro (GenePharma, Suzhou, China) | GenePharma, Suzhou, China) | 121488 |
| LV5-EF1α-mSYK-GFP&Puro (GenePharma, Suzhou, China) | GenePharma, Suzhou, China) | 123508 |
|  |  |  |
| Biological samples |  |  |
|  |  |  |
|  |  |  |
| Chemicals, peptides, and recombinant proteins | | |
| DNase I, Bovine pancreas | MCE | HY-108882 |
| Evans Blue | Sigma-Aldrich | E2129 |
| Invitrogen™ SYTOX™ Green Nucleic Acid Stains | Invitrogen™ | S7020 |
| Actinomycin D（5mg） | APE Xbio |  |
| STM2457 | Storm Therapeutic | S9870 |
| R406 | MCE | HY-12067 |
| IKKγ NBD Inhibitory Peptide TFA | MCE | HY-P1847A |
| LipofectamineTM 3000 | Invitrogen | L3000015 |
| Lipopolysaccharide | Sigma-Aldrich | L2880 |
| Nigericin sodium salt | MCE | HY-100381 |
| Collagenase, Type 1 | Sigma-Aldrich | SCR03 |
|  |  |  |
| Critical commercial assays | | |
| Neutrophil Isolation Kit, mouse | Miltenyi Biotec | S0033S |
| Reactive Oxygen Species Assay Kit | [Beyotime](https://www.baidu.com/link?url=spwu3DrxDc-_AJLs6Qg-gZN6isqlx7fcMbvhoM1C-JLlXgGVvNqN2GO7tQvFN5Dm&wd=&eqid=ed17140d00012d450000000667663a88) | MCX120 |
| Myeloperoxidase (MPO) Activity Assay Kit | Solarbio | BC5715 |
| EpiQuik CUT&RUN m6A RNA Enrichment (MeRIP) Kit Epigentek | A&D Technology Corporation | A-P-9018-24 |
| Dual-Luciferase Reporter Assay System (Promega, E1910) | Promega, E1910 | Promega, E1910 |
| Phorbol 12-myristate 13-acetate (Synonyms: Phorbol myristate acetate; PMA) | MCE | HY-18739 |
| Click-iT™ Nascent RNA Capture Kit, for gene expression analysis | Invitrogen™ | C10365 |
| Mouse MPO-DNA ELISA Kit | MEIMIAN | MM-1177M2 |
|  |  |  |
| Deposited data | | |
| MeRIP-seq | This paper |  |
|  |  |  |
| Experimental models: Cell lines | | |
| Human: HL-60 | Procell Life Science & Technology Co., Ltd | CL-0110 |
| Human: 293T/17 | Procell Life Science & Technology Co., Ltd | CL-0469 |
|  |  |  |
| Experimental models: Organisms/strains | | |
| C57BL/6mice | GemPharmatech^M^, China |  |
| Mettl3flox/flox C57BL/6 mice | Chinese Academy of Sciences University |  |
| Ly6G-Cre C57BL/6 mice | Cyagen Biosciences Inc. |  |
| Lyzm-Cre C57BL/6 mice | Jackson Laboratory |  |
|  |  |  |
| Oligonucleotides | | |
| WT-3′UTR (SYK-WT) transcript sequence, see Table S1 | This paper |  |
| MUT-3′UTR (SYK-MUT) transcript sequence, see Table S1 | This paper |  |
| qPCR primers for indicated genes, see Table S1 | This paper |  |
| MeRIP qPCR primers for indicated genes, see Table S1 |  |  |
|  |  |  |
| Recombinant DNA | | |
|  |  |  |
|  |  |  |
| Software and algorithms | | |
| GraphPad Prism 9 | GraphPad Software | http://www.bitplane.com/imaris/imaris |
| FlowJo 10 | TreeStar | http://flowjo.com/ |
| SnapGene | SnapGene Software | [https://www.snapgene.com](https://www.snapgene.com/) |
| Adobe Photoshop CC | Adobe Photoshop | <http://ps.chenqix.cn/?bd_vid=8420984592824528360> |
|  |  |  |
| Other | | |
|  |  |  |
|  |  |  |
|  |  |  |
|  |  |  |
|  |  |  |
